# Redox-dependent chaperone/peroxidase function of 2-Cys-Prx from the cyanobacterium *Anabaena* PCC7120: role in oxidative stress tolerance

**DOI:** 10.1186/s12870-015-0444-2

**Published:** 2015-02-21

**Authors:** Manisha Banerjee, Dhiman Chakravarty, Anand Ballal

**Affiliations:** Molecular Biology Division, Bhabha Atomic Research Centre, Mumbai, 400085 India

## Abstract

**Background:**

Cyanobacteria, progenitors of plant chloroplasts, provide a suitable model system for plants to study adaptation towards different abiotic stresses. Genome of the filamentous, heterocystous, nitrogen-fixing cyanobacterium *Anabaena* PCC7120 harbours a single gene (*alr4641*) encoding a typical 2-Cys-Peroxiredoxins (2-Cys-Prxs). 2-Cys-Prxs are thiol-based peroxidases that also function as molecular chaperones in plants and other systems. The Alr4641 protein from *Anabaena* PCC7120 shows high level biochemical similarities with the plant 2-Cys-Prx. The physiological role played by the Alr4641 protein in *Anabaena* was addressed in this study.

**Results:**

In *Anabaena* PCC7120, *alr4641* transcript /Alr4641 protein was induced in response to abiotic stresses and its promoter was active in the vegetative cells as well as heterocysts. The wild-type Alr4641 protein or Alr4641 lacking the peroxidatic cysteine (Alr4641C56S) or the resolving cysteine (Alr4641C178S) existed as higher oligomers in their native form. The wild-type or the mutant Alr4641 proteins showed similar chaperone activity, but only the wild-type protein exhibited peroxidase activity indicating that unlike peroxidase activity, chaperone activity was not dependent on cysteines. In contrast to other 2-Cys-Prxs, chaperone/peroxidase activity of Alr4641 was dependent on its redox state and not oligomerization status. Alr4641 could protect plasmid DNA from oxidative damage and physically associate with NADPH-dependent thioredoxin reductase (NTRC). Like 2-Cys-Prxs from plants (e.g. rice), Alr4641 could detoxify various peroxides using NTRC as reductant. On exposure to H_2_O_2_, recombinant *Anabaena* PCC7120 strain over-expressing Alr4641 (An4641^+^) showed reduced content of reactive oxygen species (ROS), intact photosynthetic functions and consequently better survival than the wild-type *Anabaena* PCC7120, indicating that Alr4641 can protect *Anabaena* from oxidative stress.

**Conclusions:**

The peroxidase/chaperone function of Alr4641, its inherent transcriptional/translational induction under different abiotic stresses and localization in both vegetative cells and heterocysts could be an adaptive strategy to battle various oxidative stresses that *Anabaena* encounters during its growth. Moreover, the recombinant *Anabaena* strain over expressing Alr4641 showed higher resistance to oxidative stress, suggesting its potential to serve as stress-tolerant biofertilizers in rice fields.

**Electronic supplementary material:**

The online version of this article (doi:10.1186/s12870-015-0444-2) contains supplementary material, which is available to authorized users.

## Background

Peroxiredoxins (Prxs) are ubiquitous peroxidases with important roles in detoxification of hydrogen peroxide, alkyl hydroperoxides and peroxynitrites [1,2]. Prxs are characterized by a conserved Alkylhydroperoxide C (AhpC) or Thiol–Specific Antioxidant (TSA) domain that contains a thioredoxin fold. Prxs have highly conserved cysteine residues, peroxidatic cysteine (Cp) and resolving cysteine (Cr), which are essential for peroxidase activity. Based on their catalytic mechanisms and the presence of conserved cysteine residues, Prxs are classified into three groups, namely, typical 2-Cys-Prx, atypical 2-Cys Prx (which are subdivided into type II Prx and PrxQ) and 1-Cys-Prx [3].

The typical 2-Cys-Prxs are functionally conserved across diverse organisms and form the largest group of peroxiredoxins. Recently, 2-Cys-Prx has been shown to be a conserved marker of circadian rhythms in all the three phylogenetic domains viz. Eukaryota, Bacteria and Archaea [4]. In typical 2-Cys-Prxs, Cp is present near N-terminus while Cr is located in the vicinity of C-terminus. On reaction with a peroxide substrate, Cp (Cys-SH) is oxidized to sulfenic acid (Cys-SOH), which in turn reacts with the thiol group of the resolving cysteine from other subunit to form an intermolecular disulfide bridge [5]. The active form of enzyme is regenerated with the help of reductants like thioredoxin. In the presence of excess substrate (e.g. H_2_O_2_), Cp of 2-Cys-Prx may undergo overoxidation to form sulfinic acid (Cys-SO_3_), which prevents disulfide bond formation, rendering the enzyme inactive. However, in many organisms, sulfiredoxin (Srx) reduces the overoxidized Cp to its catalytically active form [6,7]. Sensitivity to overoxidation depends on the structural motifs, GG(L/V/I)G and YF, which are believed to be present in the eukaryotic 2-Cys-Prxs, but generally absent in the corresponding prokaryotic enzymes [8].

The typical 2-Cys-Prx plays a vital role in detoxifying peroxides in all the kingdoms of life. Transgenic *Arabidopsis* with decreased 2-Cys-Prx in chloroplast showed oxidative damage of chloroplastid proteins indicating that 2-Cys-Prx protects the photosynthetic machinery from oxidative damage [9,10]. Also, *Arabidopsis* mutant lacking both the chloroplastid 2-Cys-Prx displayed altered redox homeostasis and showed increased H_2_O_2_ levels in leaves [11]. Overexpression of 2-Cys-Prx has been shown to protect potato plants from oxidative stress and high temperature [12]. In tobacco, the chloroplastid 2-Cys-Prx has been implicated in protecting cells from photoinhibition following exposure to high light, methyl viologen (MV) or t-butyl hydroperoxide [13]. Disruption of gene encoding 2-Cys-Prx in *Synechocystis* as well as in *Synechococcus* eliminated tolerance against H_2_O_2_ [14,15]. In bacteria like *Sulfolobus solfataricus* and *Vibrio vulnificus*, 2-Cys-Prx has been proposed to detoxify endogenously generated hydrogen peroxide, thus, supporting its role as an anti-oxidative stress protein [16,17].

Interestingly, the typical 2-Cys-Prx not only defends cells from oxidative stress, but also functions as redox-regulated chaperone depending on its oligomerization status [18]. The 2-Cys Prx from *Pseudomonas aeruginosa*, on exposure to H_2_O_2_, converts into a low molecular weight (LMW) form from its high molecular weight (HMW) form. This change triggers a chaperone to peroxidase functional switch [19]. In case of 2-Cys Prx from yeast, oxidative stress and heat shock triggers conversion from LMW form to HMW structure, which shows chaperone activity [20]. In stroma of chloroplast, under conditions of stress, the dimeric 2-Cys-Prx switches to its oligomeric form and binds reversibly to the thylakoid membrane [21]. It is widely believed that the dimeric form of 2-Cys-Prx shows peroxidatic functions while oligomerization is essential for chaperone activity [2].

Cyanobacteria, progenitors of plant chloroplasts, were the first organisms to produce oxygen as a by-product of photosynthesis [22,23]. Hence, it is expected that these organisms would have developed elaborate mechanisms to overcome oxidative stress. Filamentous forms of nitrogen-fixing cyanobacteria (e.g. *Anabaena*) are economically important as biofertilizers during cultivation of paddy in Southeast Asia [24]. *Anabaena* PCC7120, a filamentous, heterocystous, diazotrophic cyanobacterium, that tolerates abiotic stresses like radiation and desiccation, has been used as a suitable model system to study the fundamental aspects of adaptive responses to various stresses including oxidative stress in our laboratory [25-29]. Genome sequence analysis has shown *Anabaena* PCC7120 to possess several peroxiredoxin genes/ORFs (e.g. *all1541*, *alr2503*, *all2375*, *all2556*, *alr3183*, *alr4404, alr4642* and *alr4641*) [30]. The Alr3183, Alr2503, All2375 and All2556 belong to PrxQ-type of peroxiredoxins, All1541 is a type II Prx, Alr4404 is a 1-Cys-Prx, Alr4642 is Prx-like, whereas Alr4641 is a typical 2-Cys-Prx [26,31].

Earlier, 2-Cys-Prx from *Anabaena* was shown to be prone to over-oxidation [8] and was found to utilize NADPH-dependent thioredoxin reductase (NTRC) as reducing agent for peroxidase activity like the 2-Cys-Prx from rice [32]. In this study, expression analysis in response to various stresses, redox dependent chaperone/peroxidase function and the role played by this enzyme in protecting *Anabaena* from oxidative stress were addressed. Along with oxidative stress, *alr4641*/Alr4641 was induced by salt/osmotic/γ-radiation stress in *Anabaena* and the Alr4641 protein was expressed in the vegetative cells as well as heterocysts. Alr4641 formed higher oligomeric complexes and showed peroxidase/chaperone function. Unlike peroxidase activity, chaperone activity of Alr4641 did not depend on the conserved cysteine residues. Interestingly, reduction of Alr4641 with DTT resulted in loss of chaperone activity whereas treatment with H_2_O_2_ inactivated peroxidase function. Over-expression of Alr4641 in *Anabaena* protected the photosynthetic machinery from H_2_O_2_-induced damage via its peroxidatic cysteine, leading to better survival than the wild-type *Anabaena*; thus, establishing its protective role in overcoming oxidative stress.

## Results

### Abiotic stresses induce *alr4641*/Alr4641 expression in *Anabaena*

**Figure 1:**
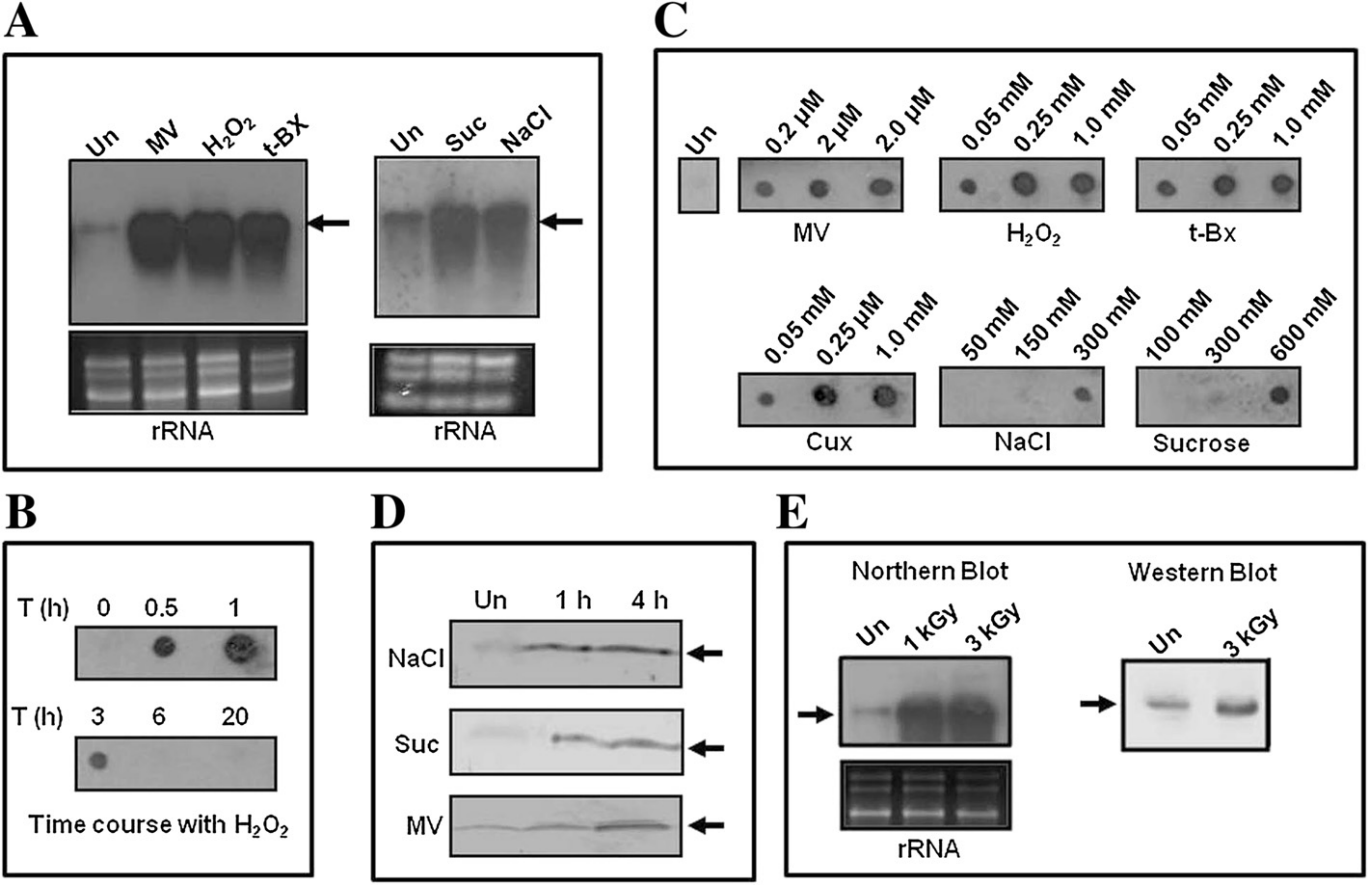
**Induction of** ***alr4641/*** **Alr4641. (A)** Northern-blotting hybridization analysis. Total RNA was isolated from *Anabaena* PCC7120 grown in BG-11 medium without any oxidative stress-causing agent (Un) or with 1 μM methyl viologen (MV), 1 mM H_2_O_2_ (H_2_O_2_), 0.25 mM t-butyl hydroperoxide (t-Bx), 50 mM NaCl (NaCl), 100 mM sucrose (Suc), resolved (5 μg per lane) on formaldehyde-agarose gels, transferred onto a nylon membrane and probed with the DIG-labeled *alr4641* ORF. The ~900-nt transcript is shown by an arrow. Blot on the left panel was exposed to the X-ray film for 30 s whereas the one on the right was exposed for 15 min. **(B)** Time course of *alr4641* expression. The wild-type *Anabaena* PCC7120 cells were treated with H_2_O_2_ (250 μM) and total RNA isolated at time points indicated. Total RNA (1 μg) from each time point was spotted on a nylon membrane and hybridized to the DIG-labeled *alr4641* probe. **(C)** The wild-type *Anabaena* PCC7120 was treated with different concentrations of MV, H_2_O_2_, t-Bx, cumeme hydroperoxide (Cux), NaCl or sucrose as indicated in the figure. Total RNA was isolated after 1 h of stress and was hybridized to the *alr4641* probe. Un, RNA from untreated control cells **(D)** Induction of the Alr4641 protein in *Anabaena*. Total proteins (20 μg per lane) were isolated from *Anabaena* cells treated with sucrose (300 mM) or NaCl (150 mM) or MV (2 μM) and probed with the Alr4641 antiserum. The 23 kD Alr4641 protein is shown by an arrow. **(E)** Total RNA isolated from untreated *Anabaena* cells (Un) or cells treated with 1 kGy or 3 kGy dose of gamma radiation was hybridized to the *alr4641* probe. After exposure to 3 kGy dose of gamma radiation, total proteins were extracted from *Anabaena* cells and probed with the Alr4641 antiserum.

Expression of *alr4641* in response to different oxidative stress inducing agents was assessed by Northern blotting-hybridization/dot blot analysis. The wild-type *Anabaena* PCC7120 cells were treated with methyl viologen (MV), hydrogen peroxide (H_2_O_2_) or tertiary butyl hydroperoxide (t-Bx) for 1 h. Subsequently, cells were harvested, total RNA isolated and probed with the *alr4641* gene probe. Results showed distinct induction of ~0.9-knt transcript in RNA isolated from *Anabaena* cells exposed to the above-mentioned oxidizing agents as compared to the untreated (control) cells (Figure 1A). Interestingly, treatment with sucrose or NaCl also enhanced the levels of the *alr4641* transcript (Figure 1A). Although, *alr4641* expression was observed as early as after 30 min of exposure to H_2_O_2_, maximal expression occurred by 1 h after which it declined and disappeared at the end of 6 h (Figure 1B). Expression profile of the *alr4641* transcript exposed to different concentrations of oxidative/osmotic stress causing agents for 1 h was monitored. With increasing concentrations, a concomitant rise in the level of the *alr4641* transcript was observed (Figure 1C). Western blotting followed by immunodetection with the Alr4641 antiserum revealed salt, sucrose and MV to increase the content of the Alr4641 protein as compared to the untreated control cells (Figure 1D). Interestingly, the *alr4641* transcript was also induced in response to ionizing (γ) radiation, a physical agent that causes oxidative stress (Figure 1E). Post irradiation, during recovery, a clear enhancement in the content of the 2-Cys-Prx protein was observed (Figure 1E).

### Alr4641 promoter is expressed in the vegetative cells as well as heterocysts

**Figure 2:**
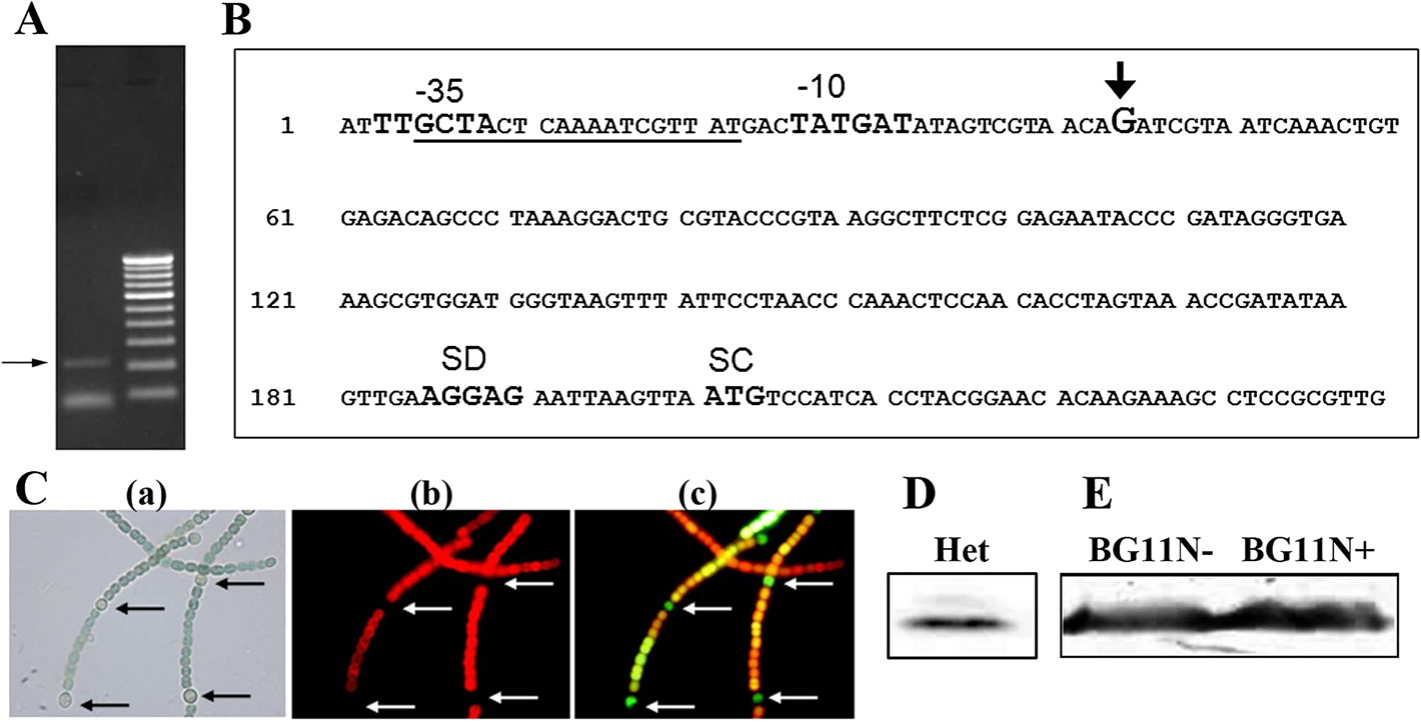
**RACE analysis and expression of the** ***alr4641*** **promoter (** ***P***_***alr4641***_ **)-** ***gfp*** **gene fusion. (A)** RACE was performed with RNA isolated from *Anabaena* cells treated with H_2_O_2_ (1 mM) for 1 h using primers described in the Methods section. The ~200-bp DNA fragment is shown by an arrow. **(B)** Sequence analysis of the RACE product. The transcriptional start site is indicated by +1 in the figure. The nucleotide sequence corresponding to the −10 and −35 region of the *alr4641* promoter, the ribosome binding site (SD) and the translational start codon (SC) are denoted while the FurA-binding sequence is underlined. **(C)** Bright field and fluorescence micrographs (1500X). An4641prom cells, were grown in medium lacking combined nitrogen for several generations and visualized under a fluorescence microscope; (a) bright field image, (b) fluorescence micrograph of cells using Hg-Arc lamp (excitation BP, 546–612 nm and emission LP, 515 nm) and (c) fluorescence micrograph (excitation BP, 450–490 nm and emission LP, 515 nm). Heterocysts are depicted by arrows. **(D)** Total protein from heterocysts (20 μg) was resolved by SDS-PAGE and probed with the Alr4641 antiserum. **(E)** Detection of the Alr4641 protein. The wild-type *Anabaena* PCC7120 cells were grown in BG-11 medium without (BG11N-) or with combined nitrogen (BG11N+). Protein extracts (60 μg per lane) were resolved by SDS-PAGE (10% gel), and immunodetected with the Alr4641 antiserum on Western blots. The 23 kD Alr4641 protein is depicted by an arrow.

As distinct induction of *alr4641* was observed in response to various abiotic stresses, it was desired to locate the *alr4641* promoter and indentify the regulatory elements associated with it. Rapid amplification of cDNA ends (RACE) with the total RNA isolated from the H_2_O_2_-treated cells showed a distinct ~200-bp cDNA product (Figure 2A). Sequence analysis of ~200-bp product identified the start of *alr4641* transcript to be 165-nt upstream of the translational start of the *alr4641* ORF (Figure 2B). Bioinformatic analysis revealed the presence of a prokaryotic −10 and −35-like promoter sequence and a putative FurA binding box within this promoter (Figure 2B). Electrophoretic mobility shift assays (EMSAs) showed the purified FurA protein from *Anabaena* PCC7120 to bind the FurA binding box (Additional file 1).

The *alr4641* promoter and its adjacent DNA were cloned upstream of the *gfp* reporter gene in reporter vector, pAM1956, and transferred into *Anabaena* PCC7120 (An4641prom). An4641prom was grown under nitrogen-fixing conditions and subjected to microscopic analysis. Interestingly, along with the vegetative cells, GFP fluorescence was also observed in the heterocysts indicating that the *alr4641* promoter was active in heterocysts as well as in the vegetative cells (Figure 2C). Moreover, the Alr4641 protein was also detected in proteins extracted from heterocysts on Western blots (Figure 2D). Expression of the Alr4641 protein was monitored in the wild-type *Anabaena* cultures grown under nitrogen-supplemented or nitrogen-fixing conditions. No significant difference in the production of Alr4641 was observed (Figure 2E) suggesting that the absence of combined nitrogen in the growth medium does not affect Alr4641 expression in *Anabaena*.

### Oligomerization of Alr4641 is independent of cysteine residues

**Figure 3:**
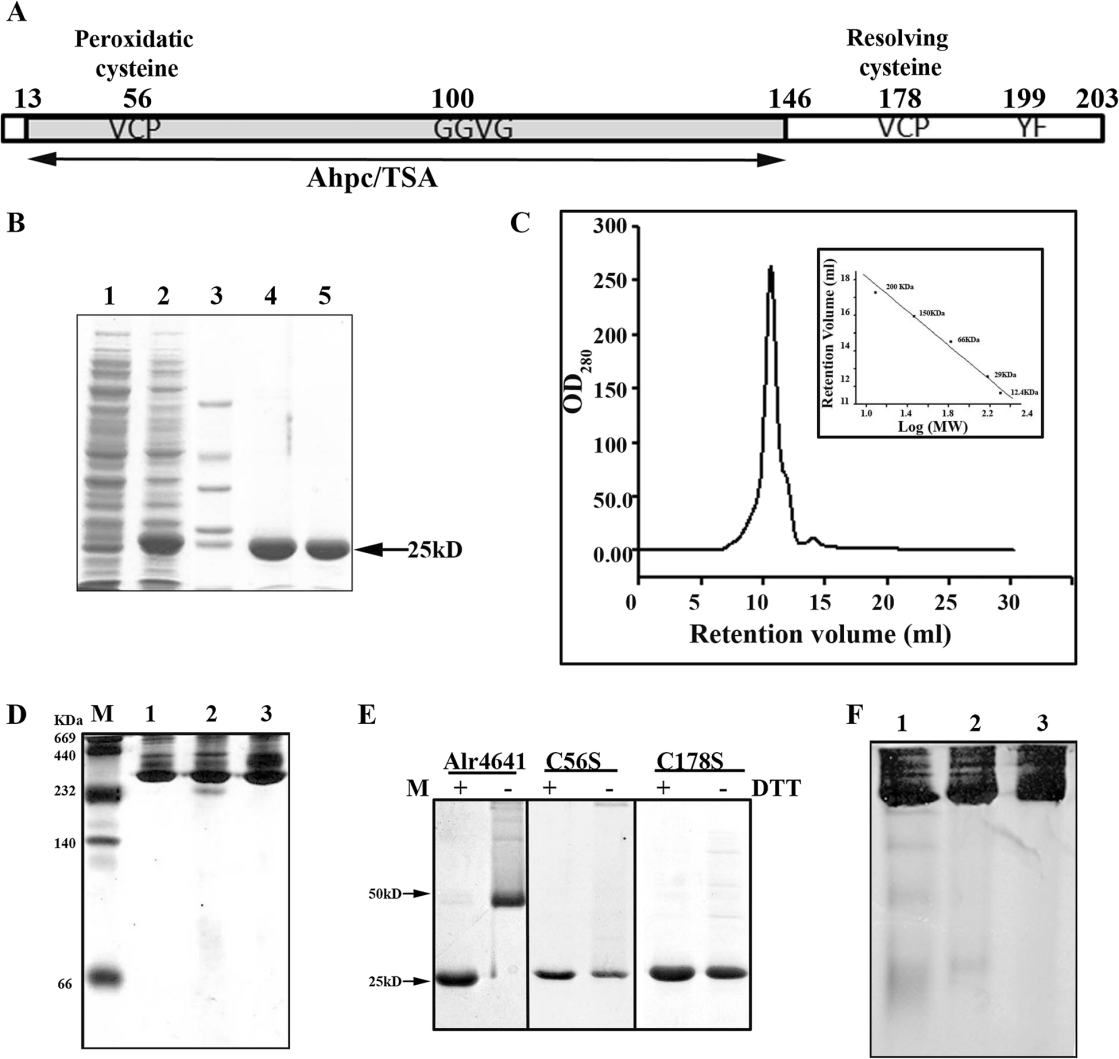
**Oligomerization of the wild-type/mutant Alr4641 proteins. (A)** *In silico* analysis. The 203 amino-acid long Alr4641 protein contains an AhpC/TSA domain at its N terminal. The amino acid residue number of the conserved VCP motif, the GGVG and YF motif, and the peroxidatic and resolving cysteines are indicated. **(B)** Purification of Alr4641 by affinity chromatography. Proteins were resolved by SDS-PAGE and visualized by staining with CBB G-250. Lane 1, whole cell protein extract (10 μg) of un-induced *E. coli* BL-21/pET4641; lane 2, whole cell protein extract (10 μg) of IPTG-induced *E. coli* BL-21/pET4641; lane3, clarified cell lysate (10 μg); lane 4, molecular mass marker (SDS-7), lane 5, 200 mM imidazole elution (7.5 μg) and lane 6, 500 mM imidazole elution (6.0 μg). **(C)** Size exclusion chromatography. The column (Superdex 200 10/300 GL) was pre-equilibrated with buffer (20 mM Tris, 50 mM NaCl, pH 7.2) and a 100μl aliquot of protein (200 μg) was injected. The retention volumes obtained with standard proteins were employed to draw a standard curve (depicted in the insert) that was used to determine the mass of Alr4641 **(D)** Native PAGE. The purified proteins (10 μg each) were resolved on native polyacrylamide gel (10%) and subsequently stained with CBB. Lane 1, native protein marker; lane 2, Alr4641; lane 3, Alr4641C56S and lane 4, Alr4641C178S. **(E)** SDS-PAGE analysis of purified proteins (each 10 μg) under reducing or non-reducing conditions. **(F)** Native PAGE of reduced or oxidized Alr4641. The Alr4641 protein (10 μg) was incubated with either H_2_O_2_ (10 mM) or DTT (5 mM) for 10 min, resolved on native polyacrylamide gels and visualized by staining with CBB. Lane 1, Alr4641 treated with H_2_O_2_ (10 mM); lane 2, untreated Alr4641 and lane 3, Alr4641 treated with DTT (5 mM).

Enhanced production of the Alr4641 protein in response to abiotic stresses and its localization in both heterocysts as well as vegetative cells suggested that Alr4641 could be an important player in detoxification of ROS in *Anabaena*. Hence, we wished to characterize the biophysical and biochemical properties of the Alr4641 protein in order to gain insights into its function. Analysis with the SMART (http://smart.embl-heidelberg.de/) or BLAST revealed the protein encoded by *alr4641* to be a typical 2-Cys-Prx containing the conserved VCP motif. The 612 bp long *alr4641* ORF encoded a 23 kD (203 amino acid) protein with the AhpC/TSA domain extending from the 13^th^ amino acid to the 146^th^ amino acid. Analysis of the Alr4641 protein sequence showed the presence of GGVG and YF motifs that are typical of eukaryotic 2-Cys-Prx (Figure 3A). Based on homology with other peroxiredoxins, cysteines at position 56 (Cys-56) and 178 (Cys-178) of Alr4641 were speculated to be the putative peroxidatic and resolving cysteine residues respectively (Figure 3A).

For functional characterization, the 2-Cys peroxiredoxin protein (Alr4641) from *Anabaena* was over-expressed in *E. coli* with N-terminal His-tag and purified near to homogeneity by affinity chromoatography (Figure 3B). The putative peroxidatic (Cys-56) and resolving (Cys-178) cysteines of Alr4641 were individually mutated to serine by site-specific mutagenesis and the corresponding proteins (Alr4641C56S and Alr4641C178S) were purified to near homogeneity. Gel filtration analysis revealed Alr4641 to elute in a fraction corresponding to decamer/dodecamer (Figure 3C). Native PAGE analysis showed the wild-type Alr4641 as well as the mutants to be present as higher oligomers (Figure 3D). These results suggest that peroxidatic and resolving cysteine residues are not involved in oligomer formation. On SDS-PAGE, the wild-type Alr4641 protein migrated as a monomer under reducing conditions (in presence of DTT), while in absence of DTT, a 50 kD protein, corresponding to its dimeric form was observed, indicating formation of inter subunit disulfide bond. Electrophoretic separation showed both Alr4641C56S and Alr4641C178S to be present as monomers irrespective of the presence or absence of reducing agent (DTT) on SDS PAGE (Figure 3E). Tryptophan fluorescence spectra of the wild-type Alr4641, Alr4641C56S and Alr4641C178S proteins showed no shift in peak position at 340 nm suggesting that the absence of Cys residues does not alter the compactness of their structure (Additional file 2). Reduction of the wild-type Alr4641 did not alter the oligomeric state of the protein, but on treatment with H_2_O_2_, few smaller oligomers along with the major higher oligomeric form were observed on native PAGE (Figure 3F). As the oligomeric state of Alr4641 remained unchanged, it was of particular interest to analyze the chaperone/peroxidase function of Alr4641 after exposure to oxidizing or reducing agents. Results pertaining to these activities are described in the next two sections.

### Alr4641 protein loses its chaperone function on reduction

**Figure 4:**
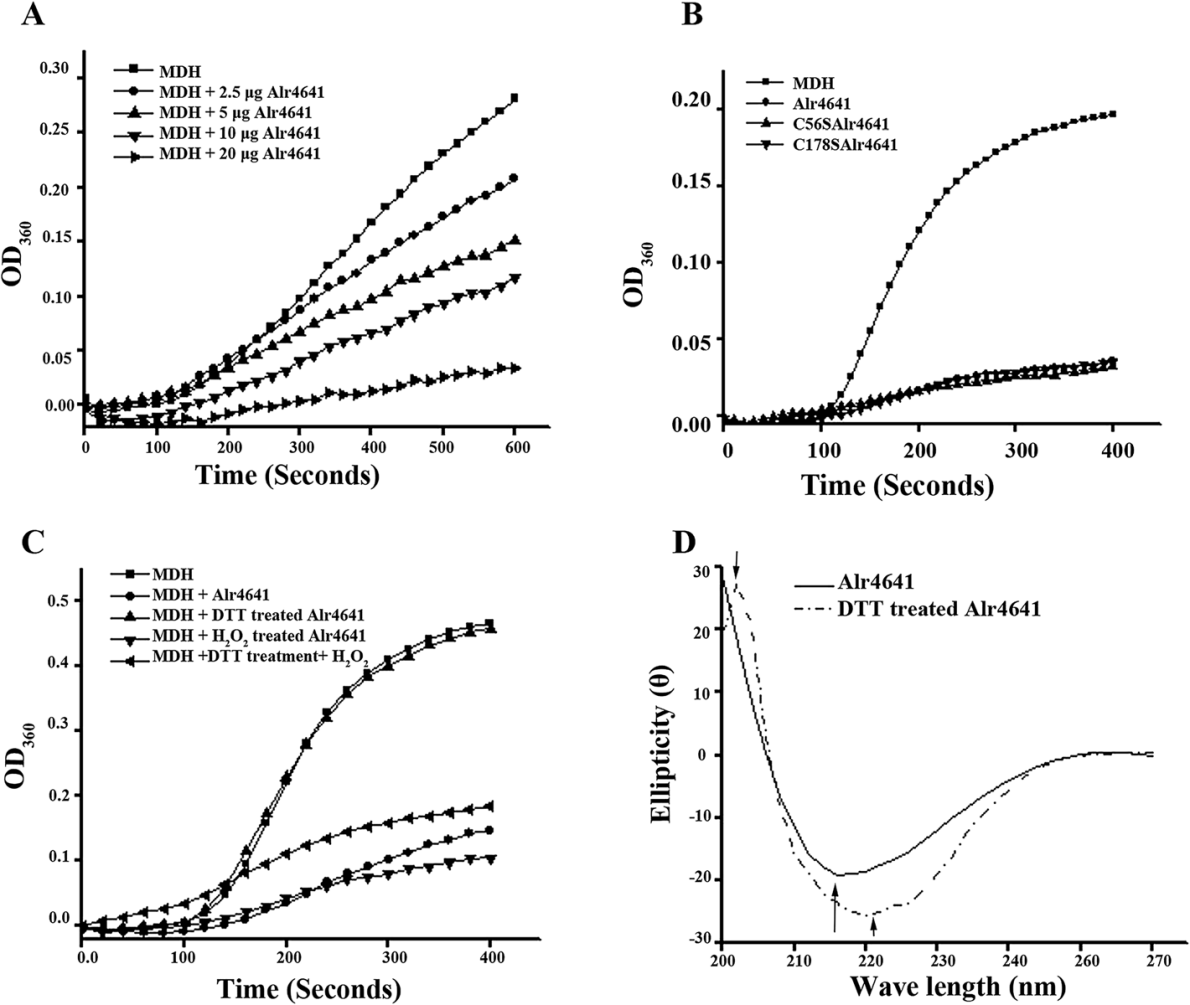
**Alr4641 functions as a molecular chaperone. (A)** Chaperone activity. Light scattering due to thermal aggregation of malate dehydrogenase (MDH, 5 μM) in the presence of different concentrations of Alr4641 (as indicated in the figure) was monitored with a spectrophotometer at 360 nm. **(B)** Light scattering of MDH was monitored (as described in A) in the presence of Alr4641C56S or Alr4641C178S or Alr4641 (20 μg each). **(C)** Chaperone activity of oxidized or reduced Alr4641. The purified Alr4641 protein was treated with H_2_O_2_ (10 mM) or DTT (10 mM) for 60 min and tested for chaperone activity with MDH (5 μM). In another reaction, the DTT-treated Alr4641 was incubated with H_2_O_2_ (5 mM) for 30 min and then employed for the chaperone assay. **(D)** Secondary structure analysis. The purified Alr4641 protein treated with DTT (10 mM) for 30 min or the control Alr4641 protein i.e. without DTT treatment (as indicated in the figure), was analyzed in a CD spectropolarimeter.

The Alr4641 protein was assessed for its capability to function as a molecular chaperone employing the malate dehydrogenase (MDH) aggregation assay. At 55°C, the MDH protein showed substantial aggregation after 10 min whereas the purified Alr4641 protein by itself did not form any aggregates. When purified Alr4641 was added to MDH, a marked decrease in the scattering of light was observed indicating reduced aggregation of MDH. Chaperone activity of Alr4641 was increased with increasing concentration of the protein (Alr4641) indicating that this protein did indeed function as a molecular chaperone (Figure 4A). Alr4641C56S and Alr4641C178S both showed chaperone activity similar to the wild-type Alr4641 (Figure 4B). Alr4641 treated with H_2_O_2_ retained chaperone activity, but interestingly, the Alr4641 protein on reduction with dithiothretol (DTT) failed to show this activity (Figure 4C). However, when the DTT-reduced Alr4641 was treated with H_2_O_2_, it regained its chaperone activity (Figure 4C). CD spectropolarimetric analysis showed significant differences in the secondary structure of the reduced and the non-reduced wild-type Alr4641 suggesting that the oxidized and reduced forms were inherently different from each other (Figure 4D).

### Alr4641 protects plasmid DNA from oxidative damage and shows Trx/NTRC-dependent peroxidase activity

**Figure 5:**
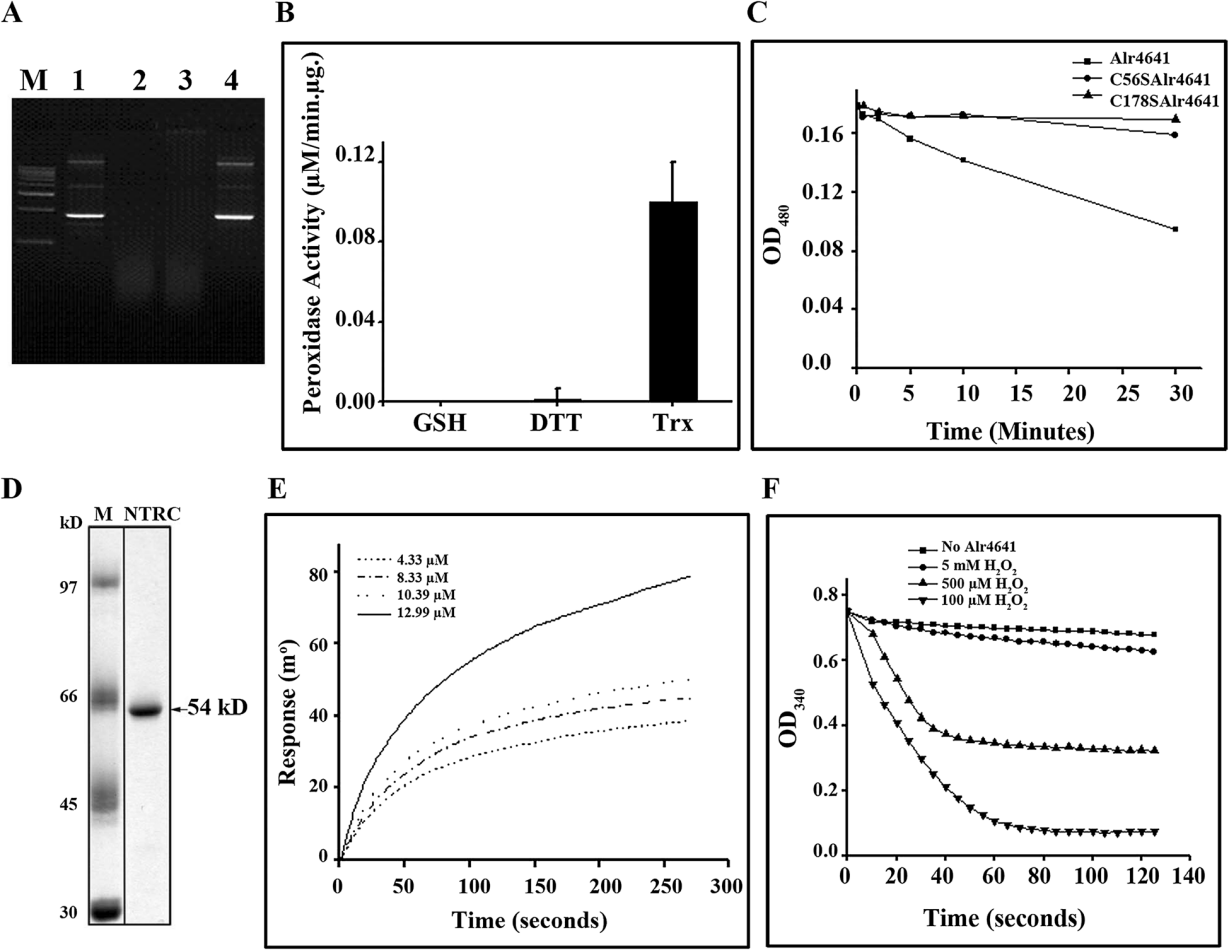
**Protection of DNA and peroxidase activity. (A)** Metal catalyzed oxidation (MCO) assay. The pBSK DNA (1 μg, lane 1) was subjected to oxidative damage using a MCO reaction (5 mM DTT + 3 μM Fe^3+^) to generate ROS in absence (lane 2) or in presence of BSA (lane 3) or purified Alr4641 (lane 4). The integrity of DNA was assessed by electrophoresis on a 1% agarose gel followed by staining with ethidium bromide. **(B)** Peroxidase activity. Relative rates of decomposition of H_2_O_2_ by the purified Alr4641 protein using various electron donors: GSH, DTT and TrxA. **(C)** Peroxidase activity of Alr4641 cysteine mutants. Decomposition of H_2_O_2_ by Alr4641C56S or Alr4641C178S or Alr4641 was monitored with 5 μM TrxA as reducing agent at different intervals of time as indicated in the figure. H_2_O_2_ was monitored as described in the Methods section. **(D)** The NTRC protein from *Anabaena* PCC7120 was over-expressed in *E. coli* and purified by affinity chromatography as described in the Methods section. After electrophoresis the proteins were visualized by staining with CBB. Lane 1, mol. mass marker and lane 2, purified NTRC protein (5 μg). **(E)** Surface plasmon resonance analysis. The Alr4641 protein was immobilized on bare gold chip utilizing the EDC-NHS chemistry (Autolab ESPIRIT User manual SPR). Different concentrations of NTRC (as indicated in the figure) were injected over Alr4641 and the response was monitored for 300 s. **(F)** Peroxidase activity of Alr4641, in the presence of NTRC, was monitored at different concentrations of H_2_O_2_ as indicated in the figure.

Metal catalyzed oxidation (MCO) was performed to verify if the purified Alr4641 protein could function as an antioxidant protein. The plasmid DNA was completely degraded when subjected to MCO assay in the absence of Alr4641. However, addition of the Alr4641 protein protected the plasmid DNA from degradation (Figure 5A). The ability of Alr4641 to scavenge hydrogen peroxide with different electron donor systems [DTT, reduced glutathione (GSH) or thioredoxin A (TrxA)] was evaluated. The Alr4641 protein could use TrxA and DTT but not GSH to detoxify H_2_O_2_ (Figure 5B). The purified Alr4641 protein showed TrxA-dependent activity whereas both Alr4641C56S and Alr4641C178S failed to do so (Figure 5C).

The NTRC protein from *Anabaena* was over-expressed in *E. coli*, purified by affinity chromatography (Figure 5D). Surface Plasmon Resonance (SPR) was employed to study interaction of Alr4641 with NTRC. The Alr4641 protein was immobilized on a bare gold sensor chip while NTRC was used in the mobile phase. The interaction between the two proteins was confirmed by a concentration-dependent increase in the SPR signal (Figure 5E). Equilibrium analysis showed a good Lorentz fit with the experimental values (Additional file 3) and the equilibrium constant (K_D_) was observed to be 1.037x10^−6^ ± 4.76x10^−8^ M. NTRC was also employed to evaluate the peroxidase activity of Alr4641 in the presence of different peroxidase substrates. Among the three substrates tested, best activity was observed with H_2_O_2_ followed by t-butyl hydroperoxide and cumeme hydroperoxide (Additional file 4). With increasing concentrations of H_2_O_2_, a reduction in the peroxidase activity was observed, indicating that excess H_2_O_2_ inactivated the Alr4641 protein (Figure 5F).

### Alr4641 forms over-oxidized monomer *in vivo* under conditions of oxidative stress

**Figure 6:**
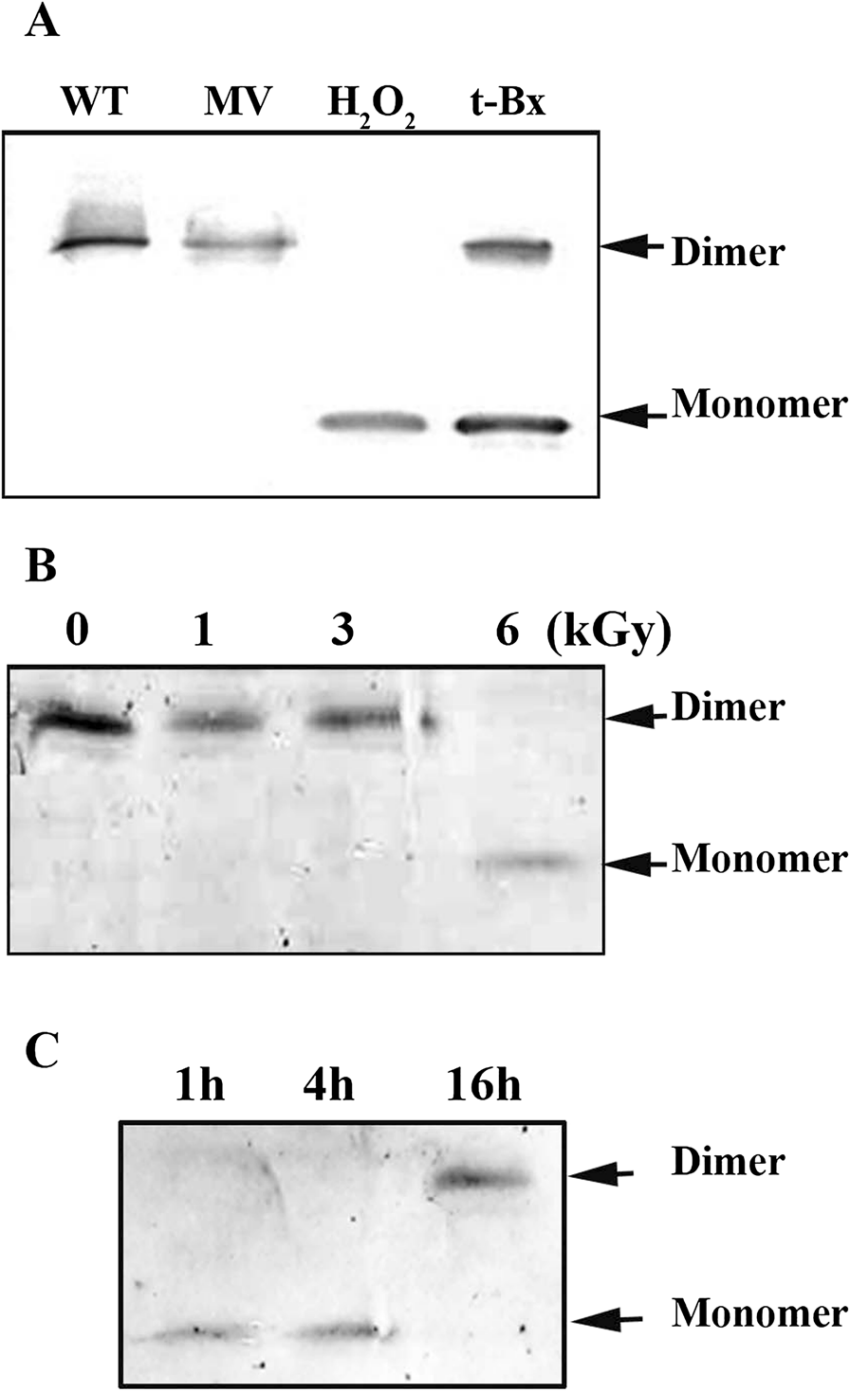
**Over-oxidation of the Alr4641 protein in** ***Anabaena*** **PCC7120. (A)** Treatment with oxidative stress-inducing agents. Exponential phase cultures of *Anabaena* PCC7120 (3.0 μg chlorophyll a ml^−1^) were exposed to methyl viologen, (MV), hydrogen peroxide, (H_2_O_2_) or t-butyl hydroperoxide, (t-Bx) for 30 min. Cell free extracts (25 μg protein per lane) were resolved on non-reducing SDS-polyacrylamide gel, electroblotted onto a nitrocellulose membrane and probed with the Alr4641 antiserum. The dimeric (non-over-oxidized) form and the monomeric (oxidized) form are indicated in the figure. **(B)** Over-oxidation of Alr4641 in response to gamma (γ) radiation. Exponential phase cultures of *Anabaena* PCC7120 (6.0 μg chlorophyll a ml^−1^) were exposed to different doses of γ-radiation as indicated in the figure. The Alr4641 protein was detected immediately after irradiation as described in A. **(C)** After exposure to 6 kGy dose of γ-radiation, *Anabaena* cells were incubated in BG11N+ medium for recovery from radiation stress. Cells were removed at time points indicated and the Alr4641 protein was detected as before.

The peroxidatic cysteine of 2-Cys-Prxs, on treatment with excess of oxidizing agents (e. g. H_2_O_2_), becomes over-oxidized and is unable to form disulphide bridges. Hence, the over-oxidized 2-Cys-Prx shows up as a monomer on non-reducing SDS-polyacrylamide gels [10]. To analyze the formation of over-oxidized monomers *in vivo* in *Anabaena* during oxidative stress, cells were treated with different oxidizing agents and analyzed (Figure 6). Treatment with H_2_O_2_ but not methyl viologen produced detectable Alr4641 monomers, whereas at the concentration of t-butyl hydroperoxide (t-Bx) employed, partial over-oxidation of Alr4641 occurred and both monomeric as well as dimeric forms of the protein were observed (Figure 6A). Exposure of *Anabaena* to 6 kGy dose of gamma radiation also resulted in the formation of the Alr4641 monomers (Figure 6B)*.* However, the over-oxidized form disappeared during recovery and 24 h after irradiation, only the dimeric form of Alr4641 was observed (Figure 6C).

### Over-expression of Alr4641, but not Alr4641C56S, causes reduction in intracellular ROS generation on exposure to H_2_O_2_

**Figure 7:**
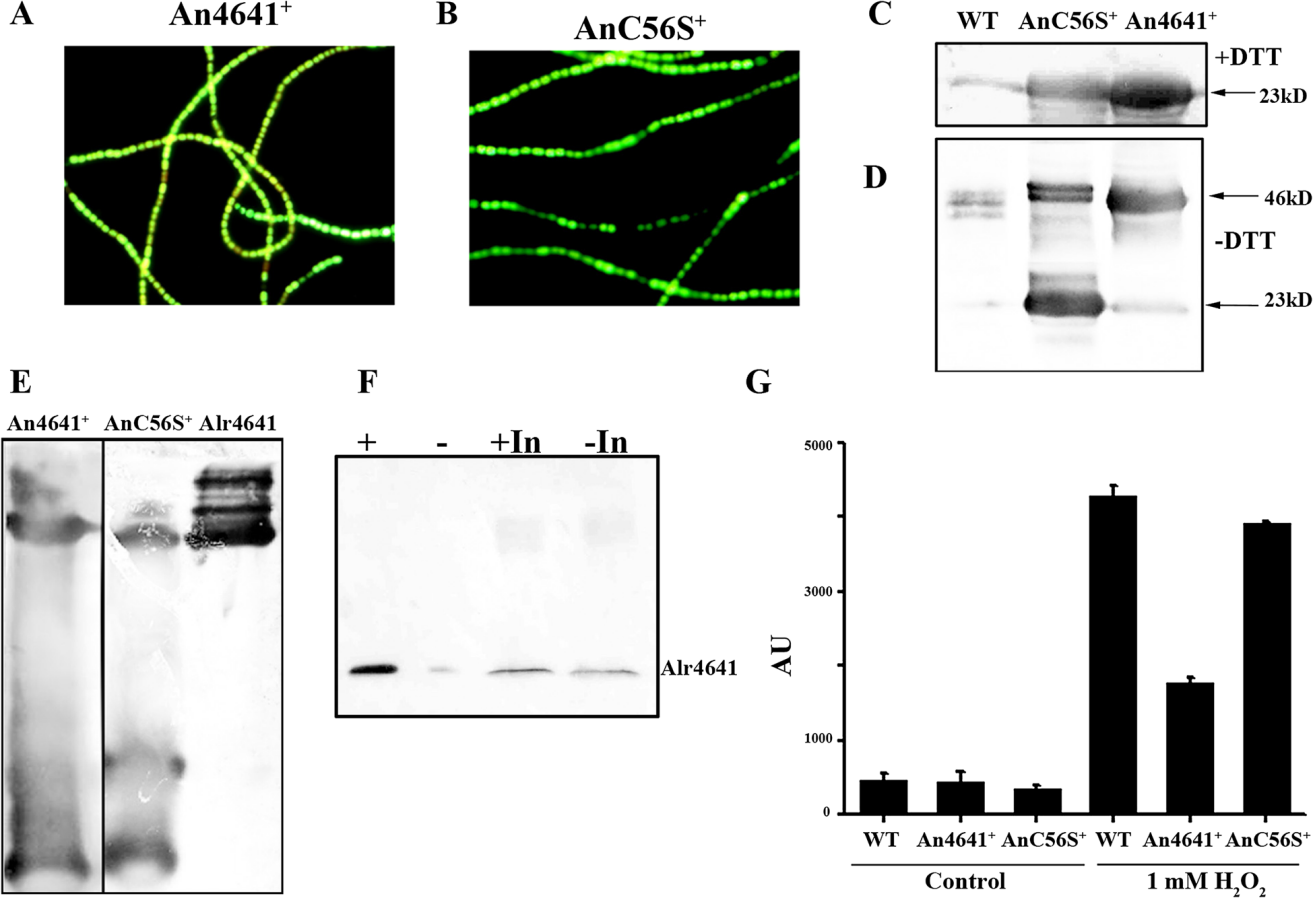
**Over-expression of Alr4641/Alr4641C56S in** ***Anabaena*** **. (A, B)** Fluorescence micrographs. The recombinant An4641^+^ **(A)** or AnC56S^+^ **(B)** cells were grown in BG-11 medium for 3 days and fluorescence microphotographs (500X magnification) using Hg-Arc lamp (excitation BP, 450–490 nm and emission LP, 515 nm) were captured. **(C, D, E)** Over-production of the Alr4641/Alr4641C56S protein in *Anabaena*. Cell-free extracts from the wild-type *Anabaena* PCC7120 (WT) or An4641^*+*^ or AnC56S^+^ (20 μg per lane) were resolved by reducing SDS-PAGE **(C)** or non-reducing SDS-PAGE **(D)** or by native polyacrylamide gel electrophoresis **(E)**. After electrophoresis, proteins were immunodetected with the Alr4641 antiserum. The Alr4641 protein is indicated by an arrow. **(F)** Physical interaction of Alr4641 with NTRC. NiNTA agarose loaded with the His-tagged NTRC protein was incubated with cell-free extract obtained from An4641^+^ (+). Unloaded NiNTA agarose (i.e. free of any bound protein) was incubated with An4641^+^ cell-free extract as negative control (−). Bound proteins were resolved on SDS-Polyacrylamide gels, transferred onto nitrocellulose membrane and probed with the anti Alr4641 antibody. Input An4641^+^ cell extract added to NiNTA agarose containing NTRC (+In) or to the negative control (−In) is also shown. **(G)** Intracellular ROS formation in response to H_2_O_2_. WT or An4641^*+*^ or cells AnC56S^+^ were grown for 3 days in BG-11 medium and treated with H_2_O_2_ (1 mM) for 1 h. Subsequently, cells were incubated with DCHFDA (10 μM final concentration) for 20 min and fluorescence emission (λ_ex_ = 490 nm, λ_em_ = 520 nm) was measured with a spectrofluorimeter. The relative fluorescence of control (untreated cells) and H_2_O_2_-treated cultures is shown in the figure.

To assess the *in vivo* role of catalytic cysteine (C56), the wild-type *alr4641 or alr4641C56S* were individually cloned between the strong light inducible *psbA* promoter (P_*psbA*_) and the *gfp* (green fluorescent protein) gene in pAM1956 (denoted as pAM4641 and pAM4641C56S respectively). Both these constructs were separately transferred to *Anabaena* PCC7120. Under fluorescence microscope, the filaments of An4641^+^ (*Anabaena* expressing Alr4641) and AnC56S^+^ (*Anabaena* over-expressing Alr4641C56S) appeared green indicating the presence of pAM4641 or pAM4641C56S (Figure 7A, B). When probed with the anti-Alr4641 antiserum, abundant production of the Alr4641 or Alr4641C56S protein was observed in the cell-free extract of An4641^*+*^ or AnC56S^+^ respectively (Figure 7C). When analyzed on non-reducing SDS-PAGE, the wild-type Alr4641 was mostly present in its dimeric form whereas Alr4641C56S remained largely monomeric (Figure 7D). Native PAGE followed by Western blot analysis with cell free extracts of An4641^*+*^/AnC56S^+^revealed the occurrence of higher oligomeric form *in vivo* in both the cases (Figure 7E) as also observed with the purified proteins (Figure 3D).

Earlier, the NTRC protein was shown to physically interact with the purified Alr4641 protein (Figure 5E). In addition, capability of NTRC to associate with the Alr4641 protein from An4641^+^ cells free extracts was assessed by pull down experiments. As shown in Figure 7F, substantial amount of Alr4641 was bound when NTRC was immobilized on NiNTA agarose. In the absence of NTRC, hardly any Alr4641 bound to the empty resin (Figure 7F).

The intracellular levels of ROS in the wild-type *Anabaena*, An4641^+^ or AnC56S^+^ cells exposed to H_2_O_2_ for 1 h were assessed with the fluorogenic probe DCHFDA. Under control conditions, ROS levels were very low in all types of cells. However, on exposure to 1 mM H_2_O_2_, substantially higher levels of ROS were observed in the wild-type *Anabaena* PCC7120 and AnC56S^+^ cells as compared to the An4641^+^ cells (Figure 7G).

### Over-production of Alr4641 protects the photosynthetic machinery and enhances survival in response to oxidative stress in *Anabaena*

**Figure 8:**
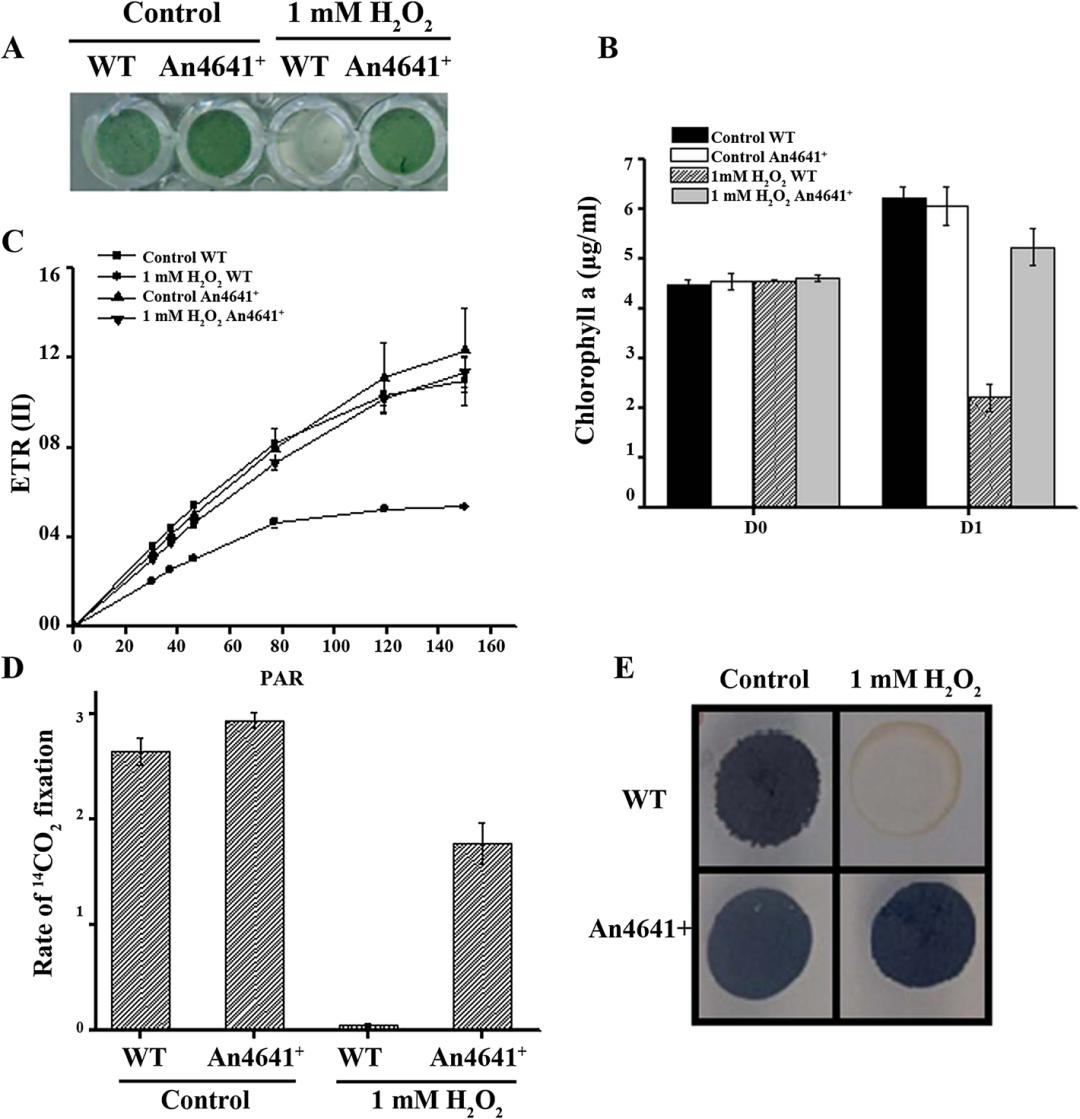
**Oxidative stress tolerance of the wild-type** ***Anabaena*** **PCC7120 (WT) and An4641** ^***+***^ **. (A)** Three-day-old *Anabaena* cultures were inoculated in a fresh growth medium and subjected to H_2_O_2_ (1 mM) stress for 2 days. Later, cultures were transferred onto a microtitre plates and photographed. **(B)** The chlorophyll *a* content of cultures shown in **(A)** was determined immediately (day 0) or after two days of exposure to H_2_O_2_. **(C)** Rapid light curves of ETR (II). Data were collected through the light response reaction from untreated (control cells) or cells treated with H_2_O_2_ (1 mM) as indicated in the figure. **(D)** The rate of ^14^CO_2_ fixation [μmoles of CO_2_ fixed (μg chlorophyll a)^−1^ h^−1^] of the wild-type *Anabaena* PCC7120 (WT) or An4641^+^ cells after treatment with 1 mM H_2_O_2_ for 24 h. **(E)** The wild-type *Anabaena* PCC7120 (WT) or An4641^+^ cells after treatment with H_2_O_2_ (1 mM) for 2 days were spotted (100 μl each) on BG-11 agar plate. The plates were incubated under continuous illumination and photographed after 14 days of incubation.

Treatment with 1 mM H_2_O_2_ for 24 h resulted in pronounced bleaching caused by a sharp reduction in the chlorophyll *a* content in the wild-type *Anabaena* PCC7120 but not in An4641^+^ cells (Figure 8A and B). A substantial decline was observed in F_v_/F_m_ of H_2_O_2_-stressed wild-type *Anabaena* cells, while An4641^*+*^ showed F_v_/F_m_ comparable to the unstressed control cells (Table 1). Light curves (LC) of electron transport rate (ETR) with the wild-type *Anabaena* or An4641^+^ were carried out to analyze electron transport rate in PSII in response to H_2_O_2_. The ETR (II) of An4641^+^ on treatment with H_2_O_2_ was similar to that of control cells. In contrast, a severe decrease in ETR (II) was observed when the wild-type *Anabaena* was treated with H_2_O_2_ (Figure 8C). Rate of CO_2_ fixation decreased marginally in the An4641^+^ treated with H_2_O_2_ as compared to a 20-fold reduction observed in the similarly treated wild-type *Anabaena* PCC7120 (Figure 8D). The wild-type *Anabaena* PCC7120 treated with H_2_O_2_ failed to grow on BG-11 plates indicating loss in viability. On the other hand, similarly treated An4641^+^ grew on plates like the unstressed control cells (Figure 8E).

**Table 1:** **PSII activity in** ***Anabaena*** **cultures treated with H**_**2**_ **O**_**2**_

**Strain (treatment)**	**F**_**v**_ **/F**_**m**_
WT (control)	0.287333 ± 0.00585
WT (H_2_O_2_)	0.195 ± 0
An4641^+^ (control)	0.286 ± 0.018358
An4641^+^ (H_2_O_2_)	0.28275 ± 0.01345

## Discussion

Prxs form a phylogenetically ancient group of enzymes with a major role in detoxification of peroxides [2]. Generally, Prxs show a moderate catalytic activity, but their high cellular content seems to compensate for their reduced efficiency in decomposing peroxides [33]. It is believed that the antioxidant system in chloroplasts, organelle with highest content of Prxs in a plant cell, has evolved from cyanobacteria. *Anabaena* bears a resemblance to plant chloroplasts in being equipped with an oxidation sensitive 2-Cys-Prx (i.e. Alr4641) along with its reducing partner NTRC [32] and showing a low catalase activity [8,25].

The presence of the 0.9-knt *alr4641* transcript (Figure 1) indicates that in spite of their adjacent location, the *alr4642* ORF (642-bp, located 191-bp downstream of *alr4641*) and *alr4641* are not co-transcribed, signifying that *alr4641* forms a monocistronic operon. The promoter sequence found immediately upstream of this start site showed the presence of FurA (a transcriptional repressor) binding site (Figure 2) and the purified FurA protein from *Anabaena* bound the DNA fragment containing this sequence (Additional file 1). Gonzalez *et al*. have shown production of the Alr4641 protein to be reduced in the FurA-overexpressing *Anabaena* PCC7120. All these results imply that FurA regulates transcription of *alr4641* in *Anabaena* [34].

In response to different abiotic stresses, the *2-Cys-prx* gene transcript was enhanced in *Synechococcus* PCC7942 but not *Synechocystis* PCC6803 suggesting that transcriptional induction of *2-Cys-prx* differs among cyanobacteria [35]. In *Anabaena*, along with methyl viologen and H_2_O_2_, gamma radiation (a physical agent that causes oxidative stress) could also enhance production of the *alr4641* transcript or the Alr4641 protein (Figure 1) [27]. Interestingly, salinity as well as osmotic stress also increased production of the Alr4641 protein (Figure 1), indicating that Alr4641 may be a multiple stress induced protein. This is not unusual as several environmental stresses (salinity, drought, heavy metals, heat shock etc.) generate ROS [12], which in turn may ultimately enhance production of the Alr4641 protein.

Employing pull down assay and SPR analysis, NTRC was shown to physically interact with Alr4641 (Figures 5E, 7F) suggesting that NTRC is likely to be the physiological reductant of Alr4641 in *Anabaena. In vivo*, on treatment with H_2_O_2_, the recombinant AnC56S^+^ strain (that over-expresses Alr4641C56S) showed higher ROS levels compared to the An4641^+^ strain (that over-expresses the wild-type Alr4641) (Figure 7G). This underscores the importance of peroxidatic cysteine of Alr4641 for detoxification of H_2_O_2_*in vivo* in *Anabaena*.

In general, the purified 2-Cys-Prx isolated from different organisms co-exists in various forms i.e. dimeric, decameric and high molecular wt. complexes [36]. In contrast, in its native form, Alr4641 as well as its cysteine mutants appeared as decamers *in vitro*, (Figure 3) or *in vivo* (Figure 7E). Moreover, irrespective of the redox state (i.e. whether oxidized or reduced), Alr4641 appeared as decamer (Figure 3) indicating that the disulfide bonds are not involved in oligomerization.

**Figure 9:**
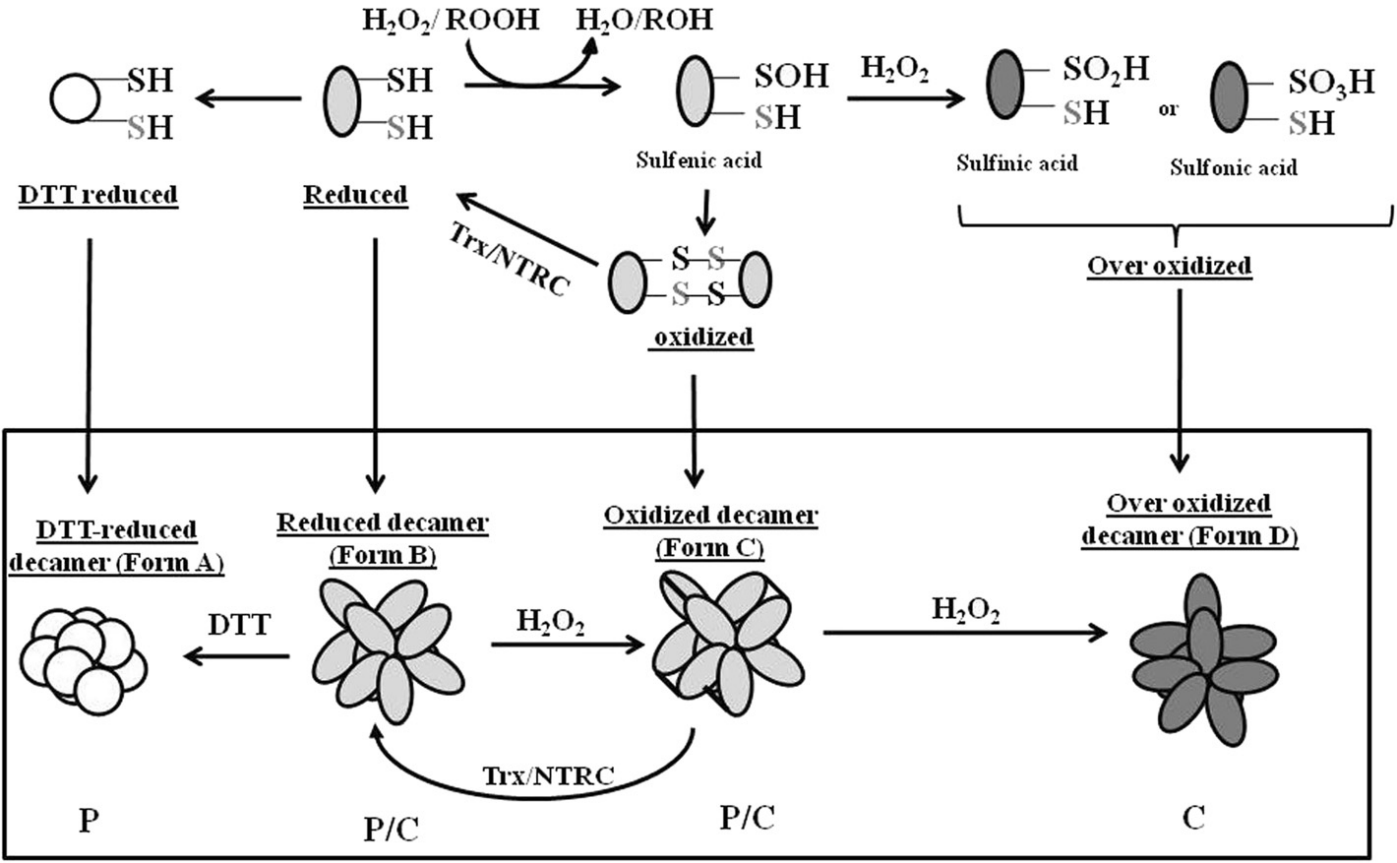
**A model depicting redox-dependent functional switching of Alr4641.** Rectangular box in the lower panel represents the oligomeric (decameric) structure of Alr4641 while the upper panel shows the state of catalytic cysteine residues of the individual monomeric units. Various conformational forms of the Alr4641 protein i.e. DTT-reduced decamer (Form-A), reduced decamer (Form-B), oxidized decamer (Form-C) and over-oxidized decamer (Form-D) are shown in the figure. Alr4641 exists as decamer irrespective of its redox state or disulphide linkage status) in all the above-mentioned conformations. Monomeric units corresponding to various forms are depicted as follows: Form-A, white circle; Form-B and Form-C, light-grey oval and Form-D, dark-gray oval. Form-B (with free thiol groups) on oxidation with H_2_O_2_ forms inter-molecular disulfide bonds (indicated with black lines in the lower panel), resulting in the formation of oxidized decamer (i.e. Form-C). Form-B is regenerated from Form-C by electron donors like Trx and NTRC. However, with excess H_2_O_2_, the cysteinyl residue of the peroxidatic cysteine of Form-B is over-oxidised to sulfinic/sulfonic acid (Form-D). Reduction of Alr4641 with DTT not only results in the loss of disulfide bonds but also changes the overall structure of the protein (Form-A). Form-B, Form-C and Form-D show chaperone activity (indicated by “C”), but Form-A fails to show this activity. On the other hand, Form-A, Form-B and Form-C function as peroxidase (indicated by “P”), whereas the over-oxidized Alr4641 i.e. Form-D is unable to do so. To summarize, under reducing conditions, Alr4641 is more likely to function as a peroxidase, whereas under oxidizing surroundings, it is more likely to work as a chaperone.

Generally, along with conformational change, the oligomeric state of 2-Cys Prx has also been linked to its dynamic redox state, which in turn determines its function [36]. Chaperone activity of 2-Cys-Prx has been generally linked to their oligomerization state [20]. The higher molecular weight complex shows chaperone function, the dimeric form mostly functions as a peroxidase, whereas the decameric form shows both these activities [37]. Although, reduction of Alr4641 with DTT did not change its oligomeric nature, its chaperone activity was severely reduced (Figure 4). Loss of chaperone function was observed on reducing Alr4641C56S and Alr4641C178S too (data not shown), suggesting that reduction in chaperone activity was not due to disruption of disulphide bonds. In contrast, treatment with H_2_O_2_ did not affect the chaperone activity of Alr4641, whereas a substantial loss in peroxidase activity was observed (Figure 5F). Apparently, chaperone/peroxidase activity of Alr4641 does not depend on its oligomeric status, but is decided by the redox state of the protein i.e. the reduced form is more likely to function as a peroxidase while the oxidized form is more liable to function as a chaperone. A model depicting the above phenomena is described in the Figure 9. Although, thought to be a specific disulphide bond reducing agent, DTT is known to affect function of proteins not containing any cysteine residues [38], and it is proposed that DTT may act as a general reducing agent that causes changes in the global structure of proteins [39]. Reduction of 2-Cys-Prx from *Arabidopsis* with DTT also resulted in altered secondary structure [37]. In this study too, a distinct difference in the CD spectra of reduced and non-reduced Alr4641 was observed (Figure 4), indicating that treatment with DTT causes a change in the secondary structure.

Excess H_2_O_2_ over-oxidizes the peroxidatic cysteine, rendering Alr4641 incapable of peroxidase activity [8]. Interestingly, high dose of γ-radiation could also over-oxidize the 2-Cys-Prx (Figure 6B) preventing the formation of disulfide bond. Post irradiation, on SDS PAGE, the monomeric form of Alr4641 disappeared and only the dimeric form could be seen after 24 h, indicating the reversion of the over-oxidised Alr4641protein to its normal form (Figure 6C). It is suggested that the recovery of the over-oxidized Alr4641 is facilitated by the sulfiredoxin (Srx) protein, which reverses the over-oxidation *in vivo* [40].

In *Anabaena*, depletion of combined nitrogen from medium leads to formation of heterocysts, the specialized cells that fix nitrogen [41]. It should be noted that in spite of lowered O_2_ content, ROS are generated by photosystem I (PSI) and respiratory electron transport in heterocysts [42]. Also, when the ratio of nitrogenase reductase to O_2_ is greater than 4, nitrogenase reductase reduces oxygen to H_2_O_2_ without being inactivated by oxygen [43]. Thus the H_2_O_2_ produced in heterocysts has to be removed promptly. Prxs are known to be differentially distributed among the vegetative cells and heterocysts [29] and among the four PrxQ-like proteins (Alr3183, Alr2503, Alr2375 and Alr2556) from *Anabaena*, only Alr2375 was detected in heterocysts [44]. In our study, promoter activity (*P*_*alr4641*_-GFP fluorescence) as well the Alr4641 protein was observed in heterocysts as well as vegetative cells (Figure 2), indicating that it may play a role in detoxifying peroxides in vegetative cells and heterocysts.

Hydrogen peroxide (H_2_O_2_) is a commonly occurring reactive oxygen species (ROS) in biological systems and cyanobacteria have been shown to be generally more sensitive to H_2_O_2_ than other phototrophs [45]. As *Anabaena* PCC7120 does not show good catalase activity [8,27], it is suggested that Prxs may be the principal components that detoxify H_2_O_2_ in this organism. H_2_O_2_ damages photosynthetic apparatus and severely affects F_v_/F_m_ in several cyanobacteria [46]. In the cyanobacterium *Microcystis aeruginosa*, treatment with H_2_O_2_ enhanced reactive oxygen species (ROS) accumulation, which caused destruction of pigment synthesis and led to cell death [47]. Treatment of the wild-type *Anabaena* PCC7120 with H_2_O_2_ caused (a) enhanced levels of ROS (b) decrease in photosynthetic activities and (c) loss in viability (Figure 8). However, all the above-mentioned deleterious effects were alleviated in An4641^+^ strain, indicating that Alr4641 can protect *Anabaena* from oxidative stress.

## Conclusions

The present study has identified Alr4641 as an abiotic stress induced protein that plays an important role in protecting *Anabaena* from oxidative stress. The Alr4641 protein was found to be unique from the other reported 2-Cys-Prxs i.e. the redox state and not its oligomerization status dictated the functional switch between the peroxidase or chaperone activity of this protein. Key attributes of Alr4641 like dual function, inherent transcriptional/translational induction under different stresses, localization in both vegetative cells and heterocysts, ability to use various reducing agents for detoxifying peroxides, reflect the versatile role played by the protein in *Anabaena*. The recombinant *Anabaena* strain over expressing Alr4641 exhibited higher tolerance to oxidative stress, thus establishing its potential to serve as stress-tolerant biofertilizers in paddy fields.

## Methods

### Organism and growth conditions

*Anabaena* PCC7120 cultures were grown in BG-11 liquid medium, pH 7.0 with combined nitrogen (17 mM NaNO_3_) under continuous illumination (30 μE m^−2^ s^−1^), with shaking (100 rpm) or without shaking (as still culture) at 27°C ± 2°C. Growth was assessed by monitoring the content of chlorophyll *a* mL^−1^ of culture volume [48]. *E. coli* cells were grown in Luria-Bertani (LB) medium in the presence of appropriate antibiotics at 37°C with shaking at 150 rpm. The antibiotics used were 10 μg neomycin mL^−1^ (Nm_10_) in BG-11 liquid media and 25 μg neomycin mL^−1^ (Nm_25_) in BG-11 agar plates for recombinant *Anabaena* PCC 7120; and 34 μg chloramphenicol mL^−1^ (Cm_34_), 50 μg kanamycin mL^−1^ (Kan_50_) or 100 μg carbenicillin mL^−1^ (Cb_100_) for *E. coli*. The *E. coli* and *Anabaena* strains and plasmids used in the study are indicated in Additional file 5.

### Cloning of *alr4641, alr4641C56S alr4641C178S, furA* and *ntrc* into pET16b

The *alr4641* ORF was PCR amplified using gene-specific primers, alr4641fwd and alr4641rev, from *Anabaena* PCC7120 chromosomal DNA (Additional file 6). The PCR product (612-bp) obtained was digested with NdeI and BamHI and cloned into similarly digested pET16b (Additional file 5) to obtain plasmid pET4641. The pET4641 insert was sequenced to confirm the nucleotide sequence integrity of the cloned gene. A point mutation, leading to substitution of the Cys codon at positions 56 or 178 to Ser codon was introduced into the *alr4641* ORF by PCR directed site-specific mutagenesis using overlapping PCR as described earlier [25]. The PCR products (*alr4641C56S* and *alr4641C178)* containing the desired mutation were cloned into pET16b vector between the NdeI and BamHI restriction enzyme sites for over-expression in *E. coli* and named pET4641C56S and pET4641C178S respectively. The *furA* (*all1691*) or the *ntrc* (*all0737*) ORF was amplified with specific primers (described in Additional file 6) employing *Anabaena* PCC7120 genomic DNA as template. Restriction enzyme sites for NcoI and BamHI were incorporated in the forward and the reverse primers respectively. The reverse primer also had six His codons (shown in bold) followed by a stop codon. PCR product was purified, digested, and ligated to the NcoI-BamHI digested pET16b vector to give rise to pETFurA and pETNTRC respectively. All the resultant clones were confirmed by DNA sequencing.

### Over-production and purification of recombinant proteins

Over-production of the His-tagged Alr4641, Alr4641C56S, Alr4641C178S, FurA and NTRC proteins in *E. coli* BL21pLysS and their subsequent purification was performed by affinity chromatography using Ni-NTA matrix as described earlier [49]. The purified Alr4641 protein was also used to immunize rabbits for generating specific antiserum. The primary and booster immunizations and collection of the antiserum were performed at a commercial facility (Merck, India).

### Size exclusion chromatography (SEC)

HPLC (AKTApure, USA) was performed using Superdex 200 10/300 GL column equilibrated at a flow rate of 0.5 ml min^−1^ at 25°C in Tris-buffer (20 mM, pH 7.2) containing NaCl (50 mM).

### Chaperone activity

The chaperone activity of purified proteins was measured by using 1 μM malate dehydrogenase (MDH) as substrate in 50 mM HEPES-NaOH (pH 8.0) buffer at 55°C with various Prx concentrations (4:1, 2:1, 1:1, 1:2 MDH:Prx molar ratio). Turbidity (A_360_) due to substrate aggregation at 55°C was monitored in a spectrophotometer (JASCO, Japan) equipped with a thermostatic cell holder. When desired, the purified Alr4641/Alr4641C56S/Alr4641C178S proteins were individually treated with DTT (5 mM) or H_2_O_2_ (10 mM) for 10 min, passed through a desalting column and employed for the chaperone assay as described above. Assays were performed at least 4 times and representative curves are shown in the figure.

### Peroxidase activity assay

For DTT dependent peroxidase assay, reaction mixture (1 ml) containing HEPES-NaOH (50 mM, pH 7.0) and desired concentrations of Prx proteins were pre-incubated with DTT (3 mM) for 10 min at 37°C, followed by the addition of H_2_O_2_ (200 μM). The reaction was stopped after 10 min by addition of TCA (10%, v/v). Subsequently, 0.2 volume of ferrous ammonium sulfate (10 mM) and 0.1 volume of potassium thiocyanate (2.5 M) were added. Absorbance of the red colored complex was spectrophotometrically measured at 480 nm. The amount of residual H_2_O_2_ remaining in the reaction was calculated from a standard calibration curve prepared by using known concentrations of H_2_O_2_. Trx-dependent peroxidase reactions were performed in a 50 μl reaction mixture containing HEPES-NaOH (50 mM, pH 7.0), *E. coli* thioredoxin A (TrxA, 5 μM), *E. coli* thioredoxin reductase (TR, 0.5 μM), NADPH (0.25 mM), Prx Protein or its mutant variants (0.05-1 μg), and H_2_O_2_ (200 μM). For GSH-dependent peroxidase activity, the typical reaction mixture contained HEPES/NaOH (50 mM, pH 7.0), NADPH (0.25 mM), glutathione reductase (GR, 0.2 μM), Prx protein (1 μg) and reduced glutathione (GSH, 5 mM). The reaction was started by addition of 100 μM peroxide substrate. The residual amount of peroxide was determined by ferrithiocyanate system as mentioned above. NTRC-dependent peroxidase activity of Alr4641 with different peroxide substrates was determined as described by Pascual *et al*. (2011) [32].

### Protein electrophoresis, Western blotting and immunodetection

Purified Alr4641, Alr4641C56S and Alr4641C178S were resolved electrophoretically by 12% SDS-PAGE with or without DTT (10 mM). These three proteins were also resolved by native PAGE and stained with CBB. Total cellular proteins from *Anabaena* cultures were extracted using Laemmli’s buffer [50] and electrophoretically separated by 12% SDS-PAGE. The gel was electroblotted on to a nitrocellulose membrane as described earlier [51]. In case of native PAGE, for efficient transfer, the gel was immersed in 1X SDS-PAGE running buffer, incubated at 70°C for 30 min and electroblotted on to the nitrocellulose membrane. Immunodetection was carried out with the Alr4641 antiserum.

### Pull down assay

In the pull-down experiment, His-tagged NTRC (500 μg) was allowed to bind to Ni-NTA agarose slurry (100 μl) in assay buffer (50 mM Tris, 200 mM NaCl, 5 mM immidazole) for 2 h at 4°C, followed by washing with same buffer to remove the unbound NTRC. Cytosolic extract of An4641^+^ (800 μg protein) was incubated with NTRC-bound Ni-NTA agarose overnight at 4°C with constant rocking. In the control experiment, only Ni-NTA agarose was incubated with cytosolic fraction of An4641^+^. In both the cases, agarose was centrifuged at 5000 g for 5 min at 4°C, washed thrice with the assay buffer, boiled with cracking buffer and resolved on 12.5% SDS-PAGE. The gel was transferred on to a nitrocellulose membrane and probed with anti-Alr4641 antibody.

### Co-Immuno-precipitation

For co-immuno-precipitation, the His-tagged NTRC (50 μg) was allowed to incubate with His-tagged Alr4641 (50 μg) in co-immunoprecipitation buffer (50 mM Tris-Cl pH 7.5, 15 mM EDTA, 100 mM NaCl, 0.1% Triton X-100 and protease inhibitor cocktail obtained from Sigma) at 4°C in duplicate. To one vial, Alr4641 antiserum was added and the components were allowed to interact for 6 h at 4°C with constant shaking. No antibody was added to the other vial (negative control). After that, a slurry of protein-G agarose beads (50 μl) was added to both the vials and these were kept shaking overnight at 4°C. Next day, beads were precipitated by centrifuging at 1200 g for 5 min at 4°C and washed thrice with the co-immuno-precipitation buffer. Subsequently, sample buffer was added to beads and the proteins extracted by boiling. The extracted proteins were separated on SDS-polyacrylamide (12.5%) gels, and visualized by staining.

### Surface plasmon resonance (SPR) analysis

Autolab Esprit SPR system was used for surface plasmon resonance analysis with bare gold sensor chip. At 20°C, about 250 response units of Alr4641 was loaded onto the bare gold chip employing the EDC-NHS chemistry (Autolab ESPIRIT User manual SPR) followed by extensive washing with buffer H (10 mM HEPES, 100 mM NaCl, pH 7.5). Different concentrations (4.33, 8.66, 10.39 and 12.99 μM) of the NTRC protein were injected onto the Alr4641-bound sensor chip at 33.3 μL min^−1^ flow rate in independent experiments. The NTRC was allowed to interact with the immobilized Alr4641 for 300 s before washing off with buffer H. The data were processed and equilibrium constant (K_D_) was calculated using Autolab kinetic evaluation software (V5.4) provided with the instrument.

### Gel retardation assays (GRA)

Primers 4641Prom_GRAFwd and 4641Prom_GRARev were annealed to form a 39-bp dsDNA (i.e. *alr4641* promoter), which was used for the gel shift assays with the purified FurA protein. The end labeling of DNA fragments with digoxygenin (DIG) and the subsequent GRA, in the presence of the non-specific competitor poly (dI-dC), was performed as described by the manufacturer (Roche).

### Metal catalyzed oxidation (MCO) assay for antioxidant activity

The plasmid DNA (pBluescript, 1 μg) was subjected to MCO by incubating FeCl_3_ (20 μM) and DTT (5 mM) at room temperature for 30 min. Purified proteins (2–20 μg) were added to the reaction mixture and further incubated for 4 h. Subsequently, DNA integrity was assessed by electrophoresis of reaction samples on agarose (0.8%, TBE, pH 7) gels.

### Oxidation and reduction of the Alr4641 protein

The purified Alr4641 protein was oxidized with H_2_O_2_ (10 mM) for 30 min or reduced by addition of DTT (5 mM). The samples were analyzed for chaperone activity as described earlier or resolved on Native PAGE and visualized by CBB staining.

### Northern blotting-hybridization and dot blot analysis

Isolation of *Anabaena* PCC7120 total RNA and subsequent Dot blot or Northern blotting-hybridization analysis with the *alr4641* DIG-labeled DNA probe was performed as described earlier [52].

### Rapid amplification of cDNA ends (RACE)

The total RNA isolated from the wild-type *Anabaena* PCC7120 cells stressed with H_2_O_2_, for 1 h was treated with DNase I and re-purified using commercial spin columns (Nucleospin RNA clean-up XS, Macherey Nagel). The reverse primer EXTERN-4641RACE-Rev (Additional file 6) was employed for cDNA synthesis. Tailing of cDNA using dATP and terminal transferase, the subsequent PCR with oligo dT-anchor primer and an internal gene-specific primer (INTER-4641RACE-Rev) was performed exactly as described (5’/3’ RACE kit, 2^nd^ Generation, Roche).

### Construction of GFP promoter

A 600-bp DNA fragment (upstream of the *alr4641* gene) that contained the *alr4641* promoter was amplified with suitable primers and cloned just upstream of *gfp* (reporter gene) in pAM1956 employing the restriction enzymes *Kpn*I and *Sac*I (construct named as pAM4641prom). This construct was conjugated into *Anabaena* PCC7120 and exconjugants (An4641 prom) were selected on BG-11/N^+^ plates containing neomycin (25 μg ml^−1^) and subjected to microscopic analysis.

### Heterocyst isolation

*Anabaena* PCC7120 was grown aerobically in nitrogen-free BG-11 liquid medium. Heterocysts were isolated from whole filaments using modified protocol as described by Cha et al., 2007 [44]. *Anabaena* culture was harvested and subjected to several freeze-thaw cycles in Tris-buffer (20 mM Tris, 1 mM EDTA, pH 7.4). After centrifugation for 5 min at 1000 g, the sedimented cells were suspended in the same buffer, glass beads (600 μM) were added and the suspension was vortexed for 2 min. Subsequently, the suspension was centrifuged at 150 g for 5 min to obtain heterocysts in the supernatant. Enrichment of greenish yellow heterocysts was achieved by repeatedly washing (7–8 times) and centrifuging (150 *g*, 5 min) the heterocyst pellet.

### Construction of pAM4641 plasmid and over-expression of Alr4641 protein in *Anabaena* PCC7120

The *alr4641* DNA fragment (~0.66-kb) from pET4641 was subcloned, downstream of the strong P_*psbA1*_ promoter, into the pFPN vector [53] employing the restriction enzymes *Nde*I and *Bam*HI (plasmid called pFPN4641). Subsequently, the *alr4641* gene along with the P_*psbA1*_ promoter was transferred as a *Sal*I–*Xma*I fragment from pFPN4641 to appropriately digested *E. coli/Anabaena* shuttle vector pAM1956 [54] to obtain pAM4641. Using a conjugal *E. coli* donor [HB101 (pRL623 + pRL443)], pAM4641 was conjugated into *Anabaena* PCC7120 as described earlier [55]. Exconjugants were selected on BG-11/N^+^ plates containing neomycin (25 μg ml^−1^) and repeatedly subcultured. The transformed *Anabaena* strain thus obtained (designated An4641^+^) was maintained on BG-11/N^+^ plates containing neomycin.

### CO_2_ fixation

The wild-type *Anabaena* PCC7120 and the recombinant An4641^+^ cells were subjected to 1 mM H_2_O_2_ treatment for 1 day. Culture aliquot (200 μl) of the above-mentioned *Anabaena* cells (5–6 μg ml^−1^ chlorophyll *a*) was incubated in white fluorescent light (24 W m^−2^) for 5 min and followed by addition of NaH^14^CO_3_ (20 mM, specific activity 0.5 mCi mmol^−1^). The reaction was stopped after 10 min by addition of 400 μl of 6 N acetic acid to the reaction mixture. The acid stable product was counted in a liquid scintillation counter with 0.4% BBOT [2, 5-bis (5-tert-butylbenzoxazole-2-yl) thiophen] dissolved in a solution containing toluene and absolute ethanol (v/v, 65:35). The experiment was performed twice with three replicate samples each time.

### DCHFDA assay

The content of the reactive oxygen species (ROS) in *Anabaena* strains treated with H_2_O_2_ for 1 day and in respective controls cells was measured with Dichlorodihydrofluorescein diacetate (DCHFDA) [56]. Briefly, DCHFDA (10 μM final concentration) was added to cells suspended in BG-11 medium (3 μg chlorophyll *a* ml^−1^). Cells were incubated for 20 min in dark at 25°C. Fluorescence emission (*λ*_ex_ = 490 nm and *λ*_em_ = 520 nm) of the control or H_2_O_2_ (1 mM)-treated cells was measured immediately afterwards. Experiments were repeated thrice and average values are reported.

### Determination of oxidative stress tolerance of An4641^+^ strain

Three-day-old *Anabaena* cultures of the wild-type *Anabaena* PCC7120 (WT) as well as An4641^+^ (in triplicates) were inoculated in a fresh growth medium at a chlorophyll *a* density of 3 μg ml^−1^ and subjected to H_2_O_2_ (1 mM) stress in tubes (without shaking) under illumination for 2 days. Growth was monitored in liquid cultures by determination of chlorophyll *a* content [48].

### Bioinformatic analysis

Amino acid sequence was analysed using BLAST (http://blast.ncbi.nlm.nih.gov/Blast.cgi) or SMART (http://smart.embl-heidelberg.de/) algorithms [57,58]. Promoter, upstream of the transcriptional start site, was identified by a promoter search program (www.softberry.com). DNA-binding consensus sequence (GATAATGATAATCA TTATC) of the *E. coli* FurA protein was used to identify corresponding sequences from DNA upstream of the *alr4641* ORF using the LALIGN program (www.ch.embnet.org).

## Additional files

Additional file 1:
**Purification of FurA and its binding with FurA binding sequence of Alr4641 promoter.** (A)The FurA protein from *Anabaena* PCC7120 was over-expressed in *E. coli* and purified by affinity chromatography as described in the Methods section. After electrophoresis proteins were visualized by staining with CBB. Lane 1, mol. mass marker and lane 2, purified FurA protein (5 μg). (B) Gel shift assays with FurA. The DNA fragment corresponding to the FurA binding site (Figure 2B) was end-labeled with DIG and employed for EMSA with the FurA protein in the presence of non-specific competitor poly (dI-dC). The samples were electrophoretically resolved, electro-blotted onto nylon membrane and probed with anti DIG antiserum. The position of DNA-protein complex is indicated by an arrow. Additional file 2:
**Tryptophan fluorescence (Ex-295nm) spectra of the wild-type Alr4641, Alr4641C56S and Alr4641C178S proteins.** Emission peaks were at the same position. Additional file 3:
**Surface plasmon resonance analysis showing interaction of Alr4641 with NTRC.** Alr4641 was loaded onto the bare gold chip employing the EDC-NHS chemistry. Different concentrations (4.33, 8.66, 10.39 and 12.99 μM) of the NTRC protein were injected onto the Alr4641-bound sensor chip at 33.3 μL/min flow rate in independent experiments. For each concentration, the experimental curve (solid lines) matches the calculated profile (dotted lines) for SPR curve. Additional file 4:
**NTRC-dependent peroxidase activity of Alr4641.** Reduction of various peroxide substrates (100 μM each, as indicated in the figure) by the Alr4641 protein in the presence of the NTRC protein was measured by monitoring the decrease in absorbance of NADPH at 340 nm. Additional file 5:
**List of** ***E. coli, Anabaena*** **strains and plasmids used in this study.**
Additional file 6:
**List of primers used in the study.**
