# Supplementary figures and images for: Redox-dependent chaperone/peroxidase function of 2-Cys-Prx from the cyanobacterium Anabaena PCC7120: role in oxidative stress tolerance
Source: BMC Plant Biol. 2015 Feb 21;15:60. doi: 10.1186/s12870-015-0444-2 (PMC4349727; doi:10.1186/s12870-015-0444-2)

## Slide 1
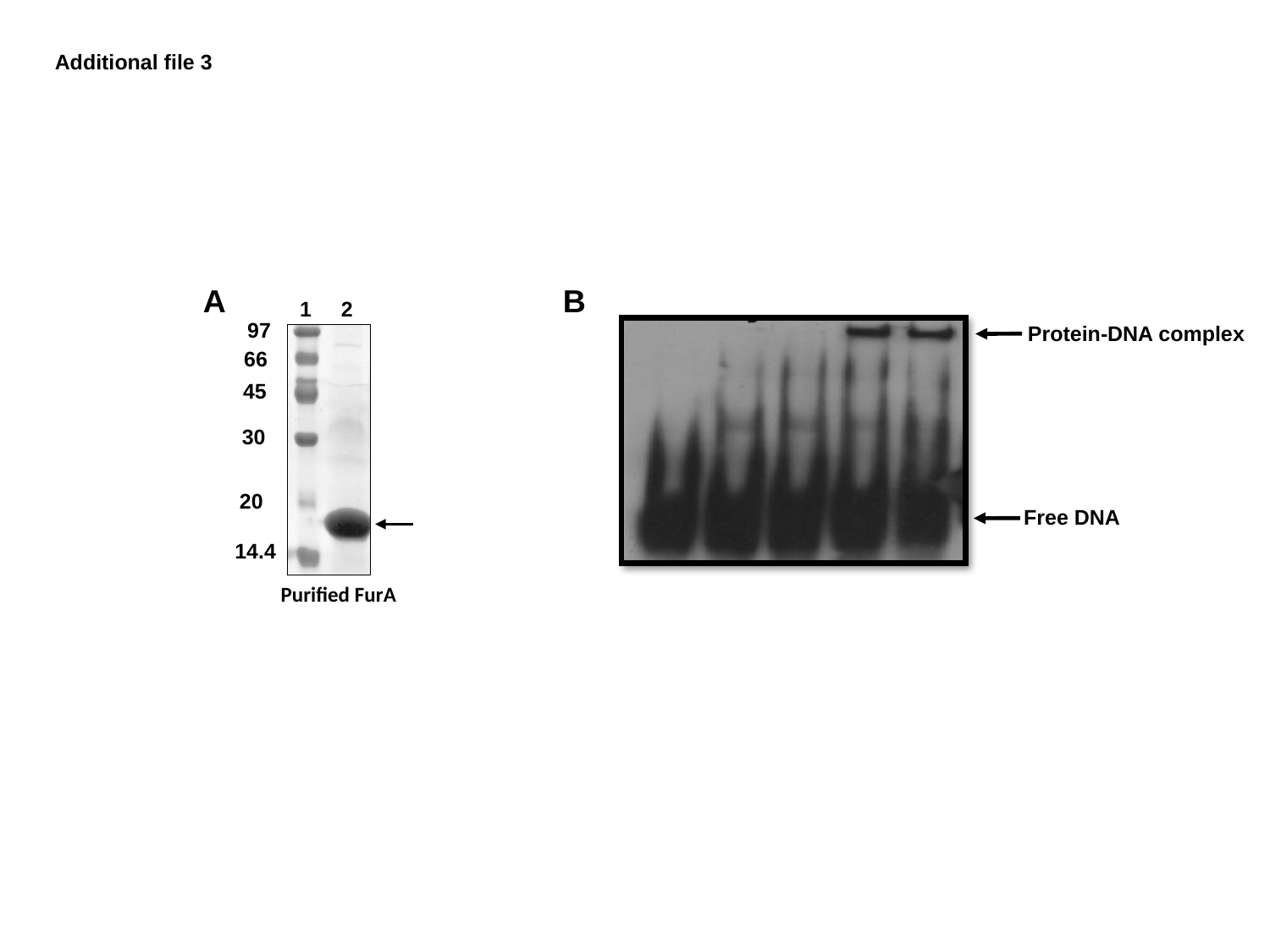

Additional file 3
A
B
1 2
97
66
45
30
20
14.4
Protein-DNA complex
Free DNA
Purified FurA

Supplement: Additional file 1: — Purification of FurA and its binding with FurA binding sequence of Alr4641 promoter. (A)The FurA protein from Anabaena PCC7120 was over-expressed in E. coli and purified by affinity chromatography as described in the Methods section. After electrophoresis proteins were visualized by staining with CBB. Lane 1, mol. mass marker and lane 2, purified FurA protein (5 μg). (B) Gel shift assays with FurA. The DNA fragment corresponding to the FurA binding site (Figure 2B) was end-labeled with DIG and employed for EMSA with the FurA protein in the presence of non-specific competitor poly (dI-dC). The samples were electrophoretically resolved, electro-blotted onto nylon membrane and probed with anti DIG antiserum. The position of DNA-protein complex is indicated by an arrow. [file 12870_2015_444_MOESM1_ESM.pptx]

## Slide 1
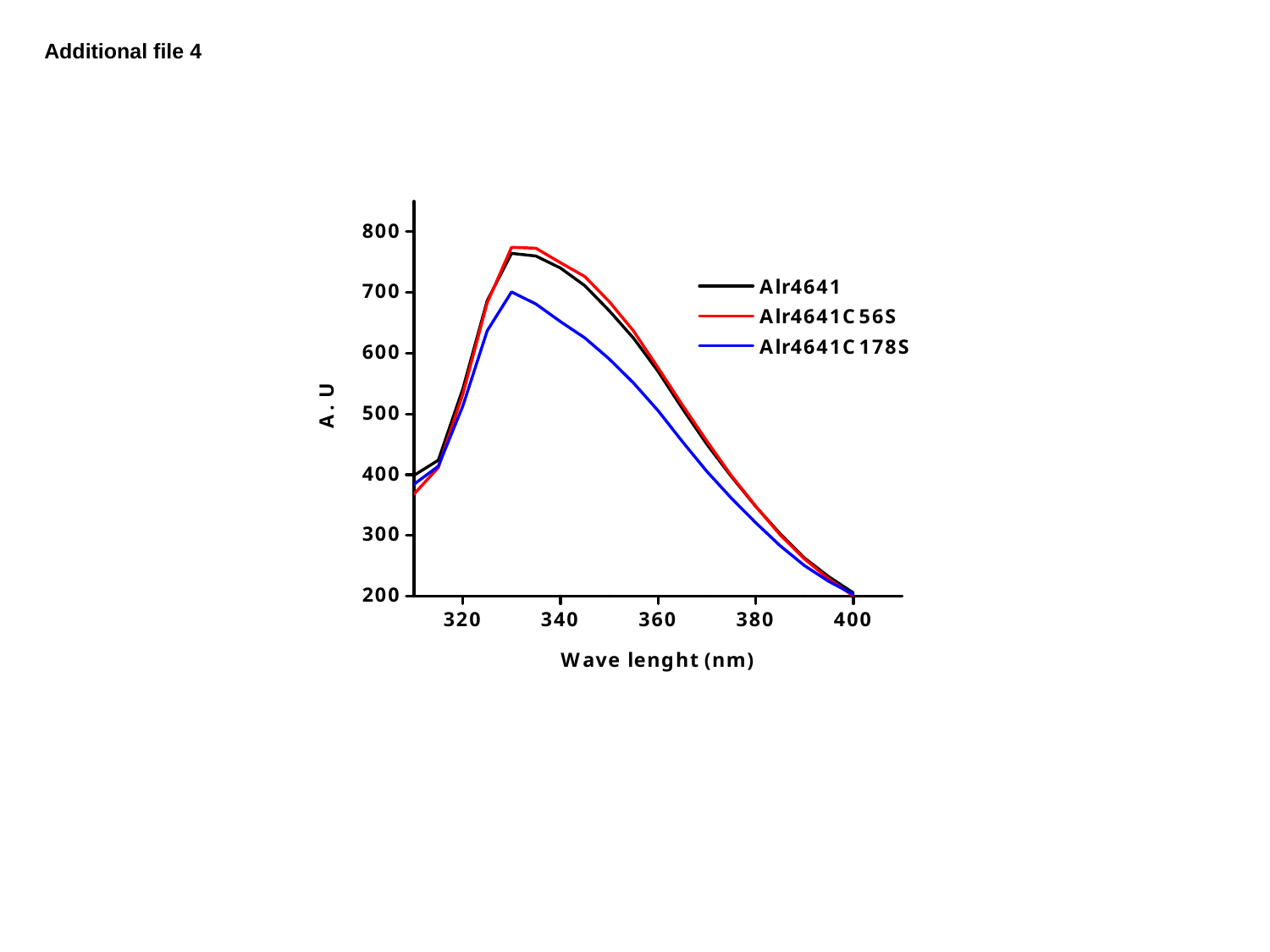

Additional file 4

Supplement: Additional file 2: — Tryptophan fluorescence (Ex-295nm) spectra of the wild-type Alr4641, Alr4641C56S and Alr4641C178S proteins. Emission peaks were at the same position. [file 12870_2015_444_MOESM2_ESM.pptx]

## Slide 1
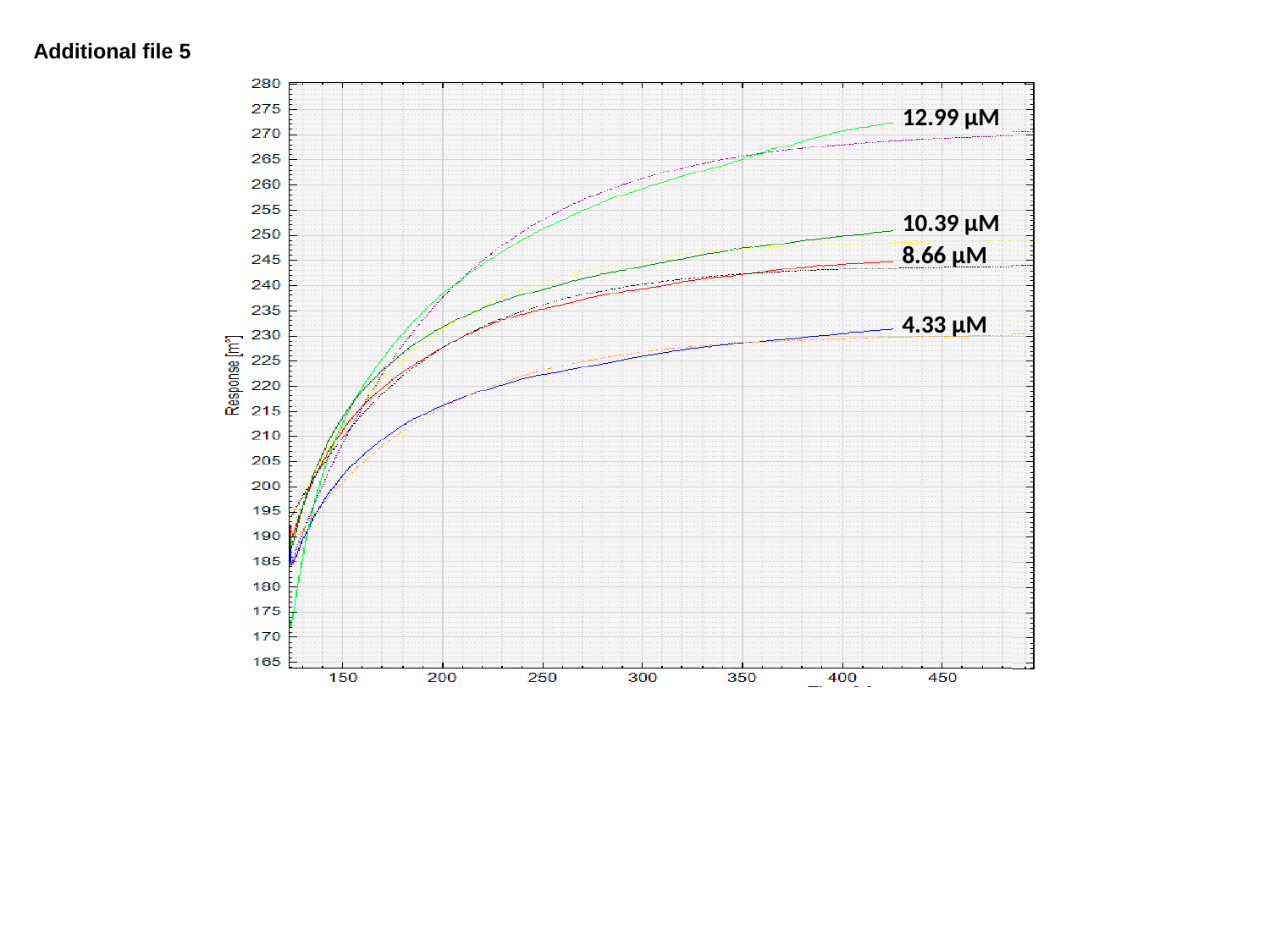

Additional file 5
12.99 μM
10.39 μM
8.66 μM
4.33 μM

Supplement: Additional file 3: — Surface plasmon resonance analysis showing interaction of Alr4641 with NTRC. Alr4641 was loaded onto the bare gold chip employing the EDC-NHS chemistry. Different concentrations (4.33, 8.66, 10.39 and 12.99 μM) of the NTRC protein were injected onto the Alr4641-bound sensor chip at 33.3 μL/min flow rate in independent experiments. For each concentration, the experimental curve (solid lines) matches the calculated profile (dotted lines) for SPR curve. [file 12870_2015_444_MOESM3_ESM.pptx]

## Slide 1
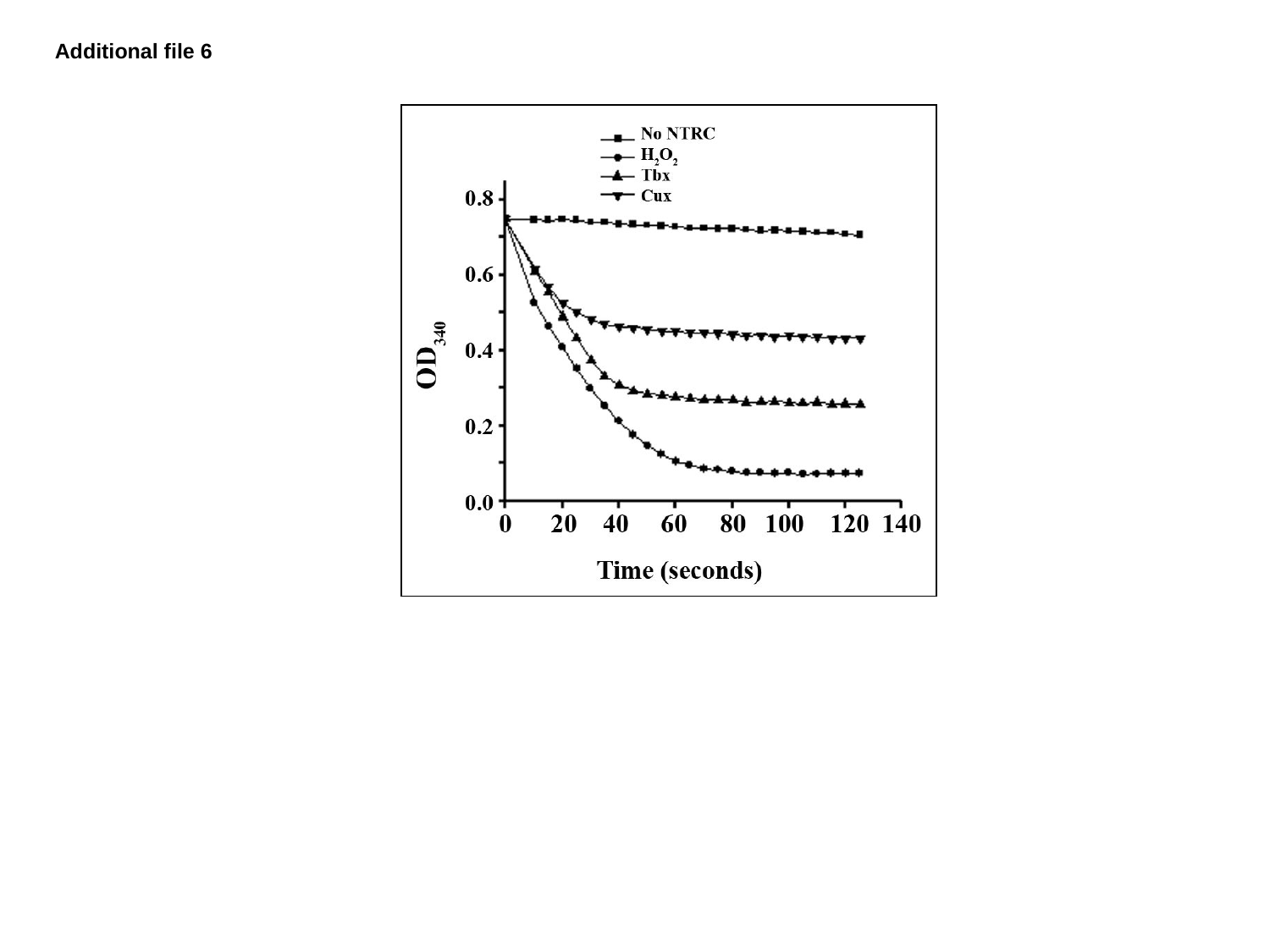

Additional file 6

Supplement: Additional file 4: — NTRC-dependent peroxidase activity of Alr4641. Reduction of various peroxide substrates (100 μM each, as indicated in the figure) by the Alr4641 protein in the presence of the NTRC protein was measured by monitoring the decrease in absorbance of NADPH at 340 nm. [file 12870_2015_444_MOESM4_ESM.pptx]
