# Supplementary material for: Redox-dependent chaperone/peroxidase function of 2-Cys-Prx from the cyanobacterium Anabaena PCC7120: role in oxidative stress tolerance
Source: BMC Plant Biol. 2015 Feb 21;15:60. doi: 10.1186/s12870-015-0444-2 (PMC4349727; doi:10.1186/s12870-015-0444-2)
Supplement: Additional file 5: — List of E. coli, Anabaena strains and plasmids used in this study. [file 12870_2015_444_MOESM5_ESM.doc]

**Additional file 5.**

**List of *E. coli, Anabaena* strains and plasmids used in this study**

| **Strains / Plasmids** | **Description** | **Source/Reference** |
| --- | --- | --- |
| ***E. coli* strains** | | |
| DH5α | F^-^ *recA*41 *endA*1 *gyrA*96 *thi*-1 *hsd*R17 (rk^-^ mk^-^) *supE*44 *relA*λ Δ*lacU*169 | Lab Collection |
| BL21(pLysS) | Cm^r^ F^_^ *ompT* h_S_ dS_B_ (r_B_^-^ m_B_^-^) *gal* *dcm* (DE3) pLysS | Novagen |
| HB 101 | F- mcrB mrr hsdS20(rB- mB-) recA13 leuB6 ara-14 proA2 lacY1 galK2 xyl-5 mtl-1 rpsL20(SmR) glnV44 λ- | Lab Collection |
| HB101R2 | Donor strain carrying pRL623(encoding methylase) and pRL443 (conjugal plasmid) | Wolk, C. P. |
| ***Anabaena* strains** | | |
| *Anabaena* PCC7120 | Wild type strain | Haselkorn, R. |
| An*4641^+^* | *Anabaena* PCC7120 harbouring pAM*4641* | This study |
| An4641Prom | *Anabaena* PCC7120 harbouring pAM*4641Prom* | This study |
| **Plasmids** | | |
| pBluescriptSKII (pBS) | Cb^r^, cloning vector | Stratagene |
| pET16b | Cb^r^, expression vector | Novagen |
| pAM1956 | Kan^r^, promoterless vector with *gfpmut2* reporter gene | Golden, S. S. |
| pFPN | Cb^r^, Kan^r^, integrative expression vector | Chaurasia et al. (2008) |
| pET4641 | 0.664 kb *alr4641* PCR product cloned into *Nde*I and BamHI sites of pET16b | This study |
| pET4641C56S | 0.664 kb *alr4641C56S* PCR product cloned into *Nde*I and *Bam*HI sites of pET16b | This study |
| pET4641C178S | 0.664 kb *alr4641C178S* PCR product cloned into *Nde*I and *Bam*HI sites of pET16b | This study |
| pETFurA | 0.456 kb FurA PCR product cloned into *Nco*I and BamHI sites of pET16b | This study |
| pETNTRC | 1.452 kb NTRC PCR product cloned into *Nco*I and *Bam*HI sites of pET16b | This study |
| pFPN4641 | 0.664 kb *alr4641* fragment cloned in pFPN at *NdeI-BamHI* restriction sites | This study |
| pAM4641 | 1.3 kb *Sma*I-*Sal*I fragment from pFPN4641 cloned in pAM1956 vector | This study |
| pAM4641Prom | 600bp *Kpn*I-*Sac*I fragment containing the *alr4641* promoter | This study |
