# Supplementary material for: Redox-dependent chaperone/peroxidase function of 2-Cys-Prx from the cyanobacterium Anabaena PCC7120: role in oxidative stress tolerance
Source: BMC Plant Biol. 2015 Feb 21;15:60. doi: 10.1186/s12870-015-0444-2 (PMC4349727; doi:10.1186/s12870-015-0444-2)
Supplement: Additional file 6: — List of primers used in the study. [file 12870_2015_444_MOESM6_ESM.doc]

**Additional file 6.**

**List of primers used in this study**

| **Primer name** | **Sequences** | **RE** |
| --- | --- | --- |
| alr4641fwd | CGGGATCCCATATGTCCATCACCTACGGAACACAAG | *NdeI* |
| alr4641rev | CGGGATCCCACAGCAGCGAAGTAAACTTTG | *BamHI* |
| 4641C56Sfwd | ACCTTTGTTTCCCCCACGGAGATC | - |
| 4641C56Srev | GATCTCCGTGGGGGAAACAAAGGT | - |
| 4641C178Sfwd | CAGATGAAGTTTCGCCTGCTGG | - |
| 4641C178Srev | CCAGCAGGCGAAACTTCATCTG | - |
| Prom4641Fwd | GGGTCGAGGAGCTCCAACCGATGGCAGAATTGAGAG | *Sac*I |
| Prom4641Rev | GGGGTACCTCTCCTTCAACTTATATCGG | *Kpn*I |
| FurAfwd | GGACCATGGCTGTCTACACAAATACTTCGCTC | *Nco*I |
| FurArev | GGGGATCCTCGAGTTAGTGATGGTGATGGTGATGAAGTGGCATCAGCGCACGTTGGC | *Bam*HI |
| NTRCfwd | GGACCATGGTTTCTTGT CACTCCCCTTG | *Nco*I |
| NTRCrev | GGGGATCCTCGAGCTAGTGATGGTGATGGTGATGAAGATTACCTTCAATCAACAGGCG | *Bam*HI |
| INTER-4641RACE-Rev | GTTCCGTAGGTGATGGACAT | - |
| EXTERN-4641RACE-Rev | AAAGTCGGGAGCCTGTTGAC | - |

Underlined sequence reflects the restriction site (RE) included in the primer
